# Isatuximab, lenalidomide, dexamethasone and bortezomib in transplant-ineligible multiple myeloma: the randomized phase 3 BENEFIT trial

**DOI:** 10.1038/s41591-024-03050-2

**Published:** 2024-06-03

**Authors:** Xavier Leleu, Cyrille Hulin, Jerome Lambert, Arthur Bobin, Aurore Perrot, Lionel Karlin, Murielle Roussel, Lydia Montes, Brieuc Cherel, Thomas Chalopin, Borhane Slama, Marie-Lorraine Chretien, Kamel Laribi, Claire Dingremont, Christophe Roul, Clara Mariette, Sophie Rigaudeau, Claire Calmettes, Mamoun Dib, Mourad Tiab, Laure Vincent, Jacques Delaunay, Alberto Santagostino, Margaret Macro, Emmanuelle Bourgeois, Frederique Orsini-Piocelle, Julie Gay, Benoit Bareau, Noemie Bigot, François Vergez, Pierre Lebreton, Reza Tabrizi, Agathe Waultier-Rascalou, Laurent Frenzel, Ronan Le Calloch, Emilie Chalayer, Thorsten Braun, Florence Lachenal, Selim Corm, Celine Kennel, Rakiba Belkhir, Jean-Sebastien Bladé, Bertrand Joly, Valentine Richez-Olivier, Helene Gardeney, Helene Demarquette, Daniela Robu-Cretu, Laurent Garderet, Muriel Newinger-Porte, Amine Kasmi, Bruno Royer, Olivier Decaux, Bertrand Arnulf, Karim Belhadj, Cyrille Touzeau, Mohamad Mohty, Salomon Manier, Philippe Moreau, Hervé Avet-Loiseau, Jill Corre, Thierry Facon

**Affiliations:** 1 Hematology, CIC 1082, U1313, CHU, University, Poitiers, France; 2 CHU, Bordeaux, France; 3 SBIM, Saint-Louis Hospital, Paris, France; 4 University Hospital, iUCT Oncopole, Toulouse, France; 5 Hôpital Lyon Sud, Pierre-Benite, France; 6 Hematology, CHU, Limoges, France; 7 Hematology, CHU, Amiens, France; 8 CH Bretagne Atlantique, Vannes, France; 9 Hematology, CHU, Tours, France; 10 Hospital center, Avignon, France; 11 Hematology, University Hospital, Inserm U1231, University of Burgundy Franche-Comté, Dijon, France; 12 Hematology, CH, Le Mans, France; 13 Internal Medicine, Tarbes-Lourdes Hospital, Tarbes, France; 14 CH La Rochelle, La Rochelle, France; 15 Hematology, CHU, Grenoble, France; 16 Hématologie – Le Chesnay, CH Versaille, Paris, France; 17 CH, Périgueux, France; 18 University Hospital, Angers, France; 19 Hematology, CH Departemental de La Roche-sur-Yon, La Roche-sur-Yon, France; 20 CHU, Montpellier, France; 21 Hôpital privé du confluent 2, Nantes, France; 22 Hematology, CH, Troyes, France; 23 Hematology, IHBN – CHU, Caen, France; 24 GHICL, Lille, France; 25 Hématologie, CH, Annecy Genevois, France; 26 Hematology, CH de la côte basque, Bayonne, France; 27 Hematology, Les Hôpitaux Privés Rennais Cesson Sévigné - Vivalto Santé, Cesson Sévigné, France; 28 Unit for Genomics in Myeloma, iUCT Oncopole, Toulouse, France; 29 Hematology, CH, Le Havre, France; 30 Hematology, CHI de Mont De Marsan, Mont-de-Marsan, France; 31 Hematology, CHU, Nîmes, France; 32 Hematology, Necker Hospital, Paris, France; 33 Hematology, CH de Cornouaille, Quimper Concarneau, Concarneau, France; 34 Hematology, CHU, Saint-Etienne, France; 35 Hematology, Avicenne hospital APHP, Bobigny, France; 36 CH Pierre Oudot, Bourgoin-Jallieu, France; 37 Medipole de Savoie, Challes les Eaux, France; 38 CH William Morey, Chalon sur Saône, France; 39 Rheumatology, Hopital Bicetre, AP-HP, Universite Paris Saclay, Paris, France; 40 Onco-Hematology Department, HIA Sainte Anne, Toulon, France; 41 CH Sud Francilien, Corbeil Essonnes, France; 42 CHU de Nice, Nice, France; 43 Hematology, CH, Dunkerque, France; 44 Hematology, CH, Lens, France; 45 Hopital Pitié Salpetriere, hématologie APHP, Paris, France; 46 Hôpital Emile Muller, Mulhouse, France; 47 DRC, CHU, Poitiers, France; 48 Hôpital Saint-Louis, Paris, France; 49 Hematology, UMR U1236, University Hospital, Rennes, France; 50 C.H.U. Henri Mondor, Créteil, France; 51 Hématologie, CHU Hotel Dieu Université, Nantes, France; 52 Hematology, Sorbonne University, Saint-Antoine Hôpital (AP-HP), UMRs 938, Paris, France; 53 Hematology, University Hospital Inserm U-S1277 and CNRS UMR9020, Lille, France

**Keywords:** Cancer, Haematological cancer, Myeloma

## Abstract

CD38-targeting immunotherapy is approved in combination with lenalidomide and dexamethasone in patients with newly diagnosed multiple myeloma (NDMM) that are transplant ineligible (TI) and is considered the best standard of care (SOC). To improve current SOC, we evaluated the added value of weekly bortezomib (V) to isatuximab plus lenalidomide and dexamethasone (IsaRd versus Isa-VRd). This Intergroupe Francophone of Myeloma phase 3 study randomized 270 patients with NDMM that were TI, aged 65–79 years, to IsaRd versus Isa-VRd arms. The primary endpoint was a minimal residual disease (MRD) negativity rate at 10^−5^ by next-generation sequencing at 18 months from randomization. Key secondary endpoints included response rates, MRD assessment rates, survival and safety. The 18-month MRD negativity rates at 10^−5^ were reported in 35 patients (26%, 95% confidence interval (CI) 19–34) in IsaRd versus 71 (53%, 95% CI 44–61) in Isa-VRd (odds ratio for MRD negativity 3.16, 95% CI 1.89–5.28, *P* < 0.0001). The MRD benefit was consistent across subgroups at 10^−5^ and 10^−6^, and was already observed at month 12. The proportion of patients with complete response or better at 18 months was higher with Isa-VRd (58% versus 33%; *P* < 0.0001), as was the proportion of MRD negativity and complete response or better (37% versus 17%; *P* = 0.0003). At a median follow-up of 23.5 months, no difference was observed for survival times (immature data). The addition of weekly bortezomib did not significantly affect the relative dose intensity of IsaRd. Isa-VRd significantly increased MRD endpoints, including the 18-month negativity rate at 10^−5^, the primary endpoint, compared with IsaRd. This study proposes Isa-VRd as a new SOC for patients with NDMM that are TI. ClinicalTrials.gov identifier: NCT04751877.

## Abstract (web summary)

In patients with newly diagnosed, transplant-ineligible multiple myeloma, addition of weekly bortzomib to isatuximab, lenalidomide and dexamethasone leads to increased minimal residual disease negativity compared with isatuximab, lenalidomide and dexamethasone.

## Main

CD38-targeting immunotherapy, daratumumab, is approved in combination with lenalidomide and dexamethasone (the triplet-based DRd regimen) in patients with newly diagnosed multiple myeloma (NDMM) that are transplant-ineligible (TI) and is considered the best SOC to date on the basis of a median progression-free survival (PFS) of 62 months^1–3^. In the MAIA study for patients with NDMM that are TI, the complete response minimal residual disease negativity 10^−5^ rate was at best 31% in the DRd arm, with a 6-month sustained MRD negativity rate at 10^−5^ of 14.9%. Novel quadruplet-based strategies are needed to further deepen responses, particularly to improve the MRD negativity rate, and to prevent relapses often responsible for early deaths in this elderly population^4–6^.

Isatuximab, an immunoglobulin (Ig) G1 monoclonal antibody, targets a specific epitope of human CD38, inducing myeloma cell death through multiple mechanisms^7,8^. Several trials have demonstrated benefit by adding isatuximab to SOC backbone regimens^2,9^. There is no report of the combination of isatuximab with Rd in patients with NDMM that are TI, although it is expected to be comparable to a DRd regimen.

Bortezomib was initially approved on a twice-weekly schedule for regimens based on 21 days^10–12^. It was shown that in patients with NDMM that are TI, including in the context of the combination of bortezomib plus lenalidomide and dexamethasone, a weekly schedule on days 1, 8 and 15 of regimens based on 28 days was safer and similarly active^13–15^. There is no report of the safety and efficacy profiles of a quadruplet-based regimen combining isatuximab with a weekly-based VRd for patients with NDMM that are TI.

The unprecedented results of MAIA in patients with NDMM that are TI favored the development of two studies for registration of the quadruplet combination of CD38-targeting immunotherapies with VRd, using daratumumab (CEPHEUS, NCT03652064) or isatuximab (IMROZ, NCT03319667) in comparison with VRd using twice-weekly bortezomib. These studies will determine the added value of CD38-targeting immunotherapy to the twice-weekly VRd SOC; however, DRd has since become a new SOC of greater efficacy based on the median PFS.

We conducted the phase 3 BENEFIT/IFM2020-05 study to demonstrate the efficacy and safety profile of the quadruplet combination CD38-targeting immunotherapy isatuximab with weekly VRd (Isa-VRd, isatuximab combined to bortezomib, lenalidomide and dexamethasone) compared with the triplet combination isatuximab with Rd (IsaRD, isatuximab combined to lenalidomide and dexamethasone) in a population of patients with NDMM that are TI. Here we report the primary analysis of BENEFIT/IFM2020-05.

## Results

### Patients and treatment

**Fig. 1:**
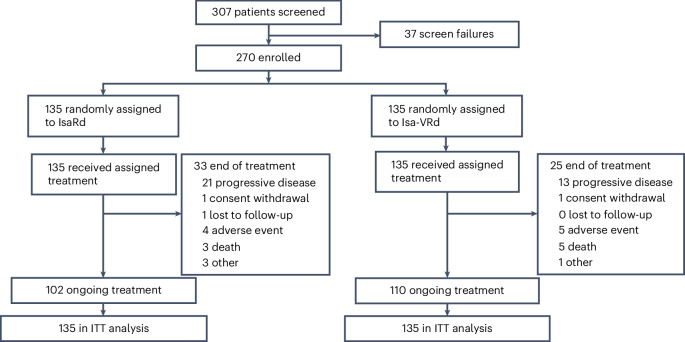
CONSORT patient flow diagram. Patient disposition at the data cutoff date of 25 March 2024.

A total of 270 patients were enrolled, with 135 assigned to either the IsaRd or Isa-VRd arm, and received at least one dose of treatment (Fig. 1). The baseline characteristics of the patients were well balanced across the study arms (Table 1).

**Table 1:** Baseline demographic and disease characteristics (ITT population^a^)

	IsaRd (*N* = 135)	Isa-VRd (*N* = 135)
**Sex,** ***n*** **(%)**		
Female	64 (47)	61 (45)
Male	71 (53)	74 (55)
**Median age (IQR), years**	73.6 (71–76)	73.2 (71–76)
**Age category,** ***n*(%)**		
<70	25 (19)	28 (21)
70–75	62 (46)	65 (48)
≥75	48 (36)	42 (31)
**ECOG performance status,** ***n*** **(%)**^b^		
0 or 1	119 (88)	125 (93)
>1	16 (12)	10 (7)
**eGFR** **<** **60** **ml per min per 1.73 m**^**2**^ **(MDRD),** ***n*** **(%)**	28 (21)	19 (14)
**Median time between diagnosis of multiple myeloma and randomization (range), months**	0.9 (0.7–1.5)	1 (0.7–1.7)
**Type of myeloma at baseline,** ***n*** **(%)**		
IgG	84 (66)	79 (62)
IgA	26 (20)	37 (29)
**Light chain only (κ and/or γ)**	16 (12)	12 (9)
**ISS stage at baseline,** ***n*** **(%)**^c^		
Stage I	51 (38)	50 (37)
Stage II	57 (42)	64 (47)
Stage III	27 (20)	21 (16)
**R-ISS stage at baseline,** ***n*** **(%)**^d^		
Stage I	35 (26)	32 (24)
Stage II	89 (66)	92 (68)
Stage III	11 (8)	11 (8)
**Cytogenetic risk at baseline,** ***n*** **(%)**^e^		
Standard	75 (60)	68 (53)
Intermediate	41 (33)	48 (37)
High	10 (8)	13 (10)

Isa-VRd denotes isatuximab plus bortezomib and lenalidomide and dexamethasone.

IsaRd denotes isatuximab and lenalidomide and dexamethasone.

^a^The intention-to-treat (ITT) population was defined as all patients who underwent randomization.

^b^Eastern Cooperative Oncology Group (ECOG) performance status is scored on a scale from 0 to 5, with 0 indicating no symptoms and higher scores indicating increasing disability.

^c^ISS disease stage is based on the combination of serum β_2_-microglobulin and albumin levels. Higher stages indicate more advanced disease.

^d^The Revised International Staging System (R-ISS) disease stage, derived on the basis of the combination of serum β_2_-microglobulin, albumin and lactate dehydrogenase levels, and genetic risk by NGS, consists of three stages, with higher stages indicating more advanced disease.

^e^Cytogenetic risk was assessed according to Perrot et al. for study analysis^28^. Bone marrow samples were obtained at diagnosis and shipped overnight to a central laboratory. Upon receipt, plasma cells were isolated using CD138^+^ magnetic-activated cell sorting (Miltenyi Biotec). Post-sorting purity was checked by cytologic analysis of a spin from a positive fraction, and only samples with ≥70% plasma cells after sorting were retained for analysis. The mean purity was 94%. Plasma cells were analyzed by NGS using NextSeq 500 (Illumina). For each positive del(17p) assessed by NGS, an additional fluorescence in situ hybridization analysis was performed to assess the percentage of positive plasma cells. NGS sequencing was performed using a panel of specific probes targeting regions of interest, as previously described^29,30^.

eGFR, estimated glomerular filtration rate; IQR, interquartile range; MDRD, modification of diet in renal disease.

At the clinical cutoff date (25 March 2024), 50 (19%) patients discontinued at least one of the study treatments, 30 in the IsaRd group and 20 in the Isa-VRd group. The most common reason for treatment discontinuation was progressive disease in 34 patients (59%): IsaRd, 21 (64%) and Isa-VRd, 13 (52%).

More precisely, 49 (18%) stopped isatuximab (29 IsaRd and 20 Isa-VRd), 49 (18%) stopped lenalidomide (29 IsaRd and 20 Isa-VRd) and 18 (13%) stopped bortezomib. The reasons for discontinuation of bortezomib are reported in Extended Data Table 1. The median relative dose intensity was 91.6% (95% CI, 82 to 96) for bortezomib, 95.8% (IsaRd, 95.8%; Isa-VRd, 96.1%) for isatuximab, 91.1% (IsaRd, 91%; Isa-VRd, 91.7%) for lenalidomide and 95.8% (IsaRd, 97.9%; Isa-VRd, 95.8%) for dexamethasone (Table 2).

**Table 2:** Duration of treatment and relative dose intensities^a^ in the safety population^b^

	IsaRd (*N* = 135)	Isa-VRd (*N* = 135)
**Isatuximab**		
Duration of treatment, months	15.8 (15.6 to 16.1)	15.9 (15.6 to 16.3)
Relative dose intensity, %	95.8 (91 to 99)	96.1 (90.9 to 99.9)
**Bortezomib**		
Duration of treatment, months	—	15.7 (13.4 to 16.3)
Relative dose intensity, %	—	91.6 (81.8 to 95.6)
**Lenalidomide**		
Duration of treatment, months	15.8 (15.6 to 16.1)	15.9 (15.6 to 16.3)
Relative dose intensity, %	91 (74.3 to 99)	91.7 (72.5 to 99.5)
**Dexamethasone**		
Duration of treatment, months	10.2 (10.1 to 10.6)	10.2 (10.1 to 10.6)
Relative dose intensity, %	97.9 (75.5 to 100)	95.8 (71.9 to 100)

Values are given as median (95% CI).

Isa-VRd denotes isatuximab plus bortezomib/lenalidomide/dexamethasone.

IsaRd denotes isatuximab /lenalidomide/dexamethasone.

^a^Dose intensity was defined as the ratio of total administered dose to total planned dose.

^b^The safety population included all patients who received at least one dose of study treatment.

### Efficacy

**Fig. 2:**
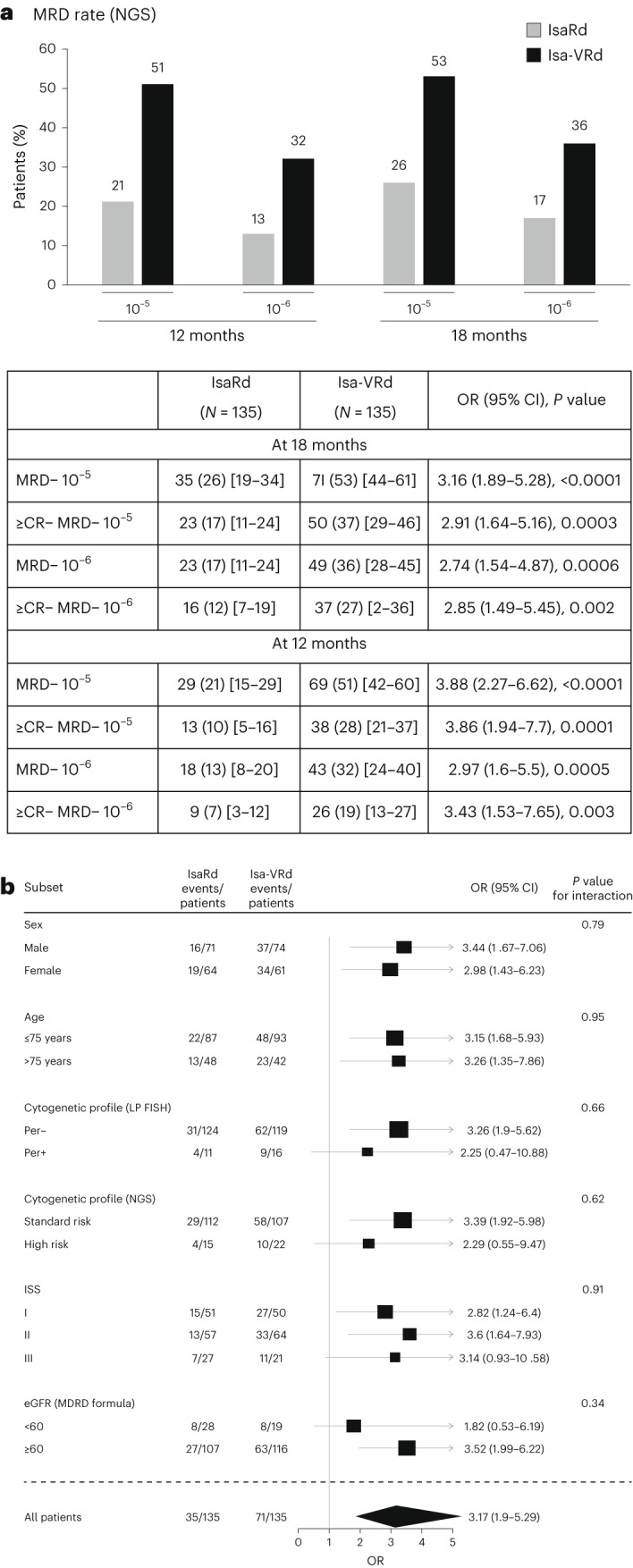
Summary of MRD status and prespecified MRD subgroup analyses. Shown are the results of MRD endpoints (**a**) and prespecified MRD subgroup analyses at 18 months (**b**). **a**, Parentheses indicate 95% CI. LP FISH, linear predictor interphase fluorescence in situ hybridization; Per−, not high-risk LP ≤ 1; Per+, high-risk score LP > 1; MRD−, MRD negativity; CR− MRD−, patients that are both in CR or better and MRD negativity.

MRD negativity at 10^−5^ at 18 months occurred in 106 (39%) patients. The 18-month MRD negativity rate at 10^−5^ was significantly higher in the Isa-VRd arm, with 71 patients (53%, 95% CI 44 to 61) compared with 35 patients (26%, 95% CI 19 to 34) in the IsaRd arm. The odds ratio (OR) for MRD negativity in the Isa-VRd group compared with the IsaRd group was 3.16 (95% CI 1.89 to 5.28, *P* < 0.0001) (Fig. 2a). The higher MRD negativity rates in Isa-VRd were also observed at 12 months at both 10^−5^ and 10^−6^. Prespecified MRD subgroup analyses confirmed a consistent benefit across most subgroups, including some difficult-to-treat populations with worse prognostic factors (Fig. 2b).

**Fig. 3:**
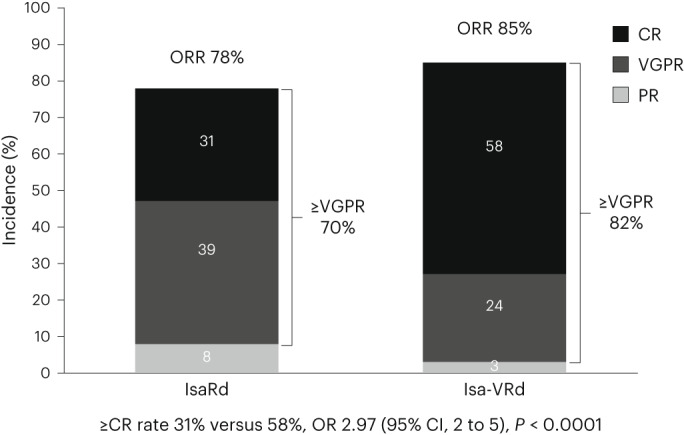
Responses in the ITT population at 18 months. ORR, overall response rate.

**Extended Data Fig. 1:**
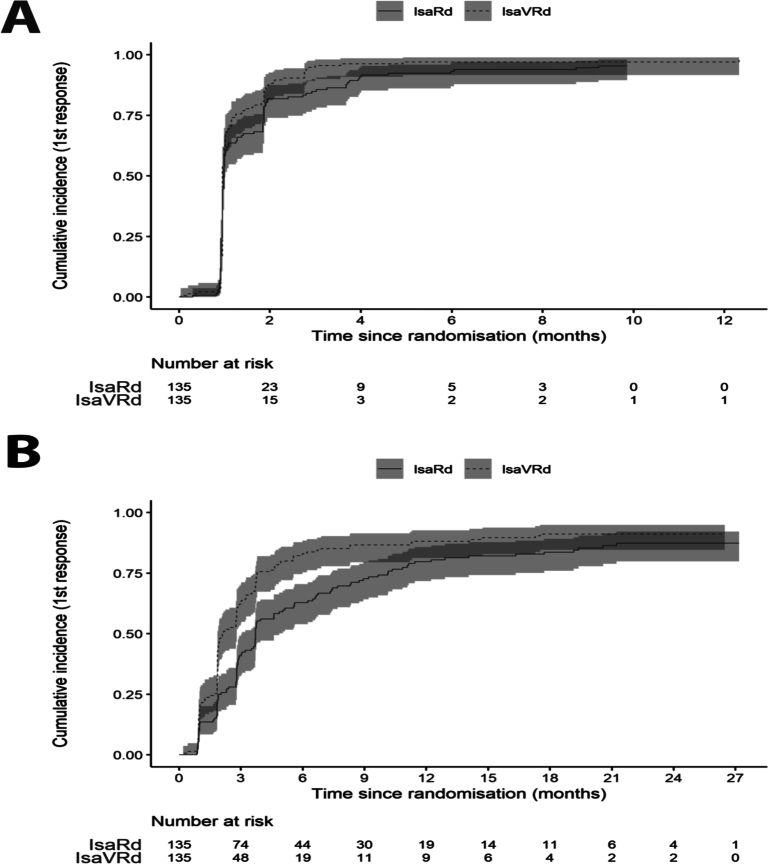
Time to the first occurrence of a confirmed response in the ITT population. (**A**) In patients with PR or better (p = 0.040) among patients in the intention-to-treat population. (**B**) In patients with VGPR or better (p=0.0002) among patients in the intention-to-treat population.

The overall rates of very good partial response or better (≥VGPR) and complete response or better (≥CR) were higher at 18 months with Isa-VRd than with IsaRd. The ≥CR rate was 58% versus 31%, OR 2.97 (95% CI 2 to 5), *P* < 0.0001 (Fig. 3). The time to the first occurrence of a confirmed at least a partial response (≥PR) and ≥VGPR were significantly shorter in IsaRd versus Isa-VRd. The median time to first occurrence for patients ≥PR were 0.99 (95% CI 0.95 to 1.02) months versus 0.95 (95% CI 0.95 to 0.99) months (hazard ratio (HR) 1.30 (95% CI 1.01 to 1.67), *P* = 0.040), and for patients ≥VGPR, 3.7 (95% CI 3 to 4.9) months and 2.1 (95% CI, 1.9 to 2.9) months (HR 1.65 (95% CI 1.27 to 2.14), *P* = 0.0002), respectively (Extended Data Fig. 1a,b).

**Extended Data Fig. 2:**
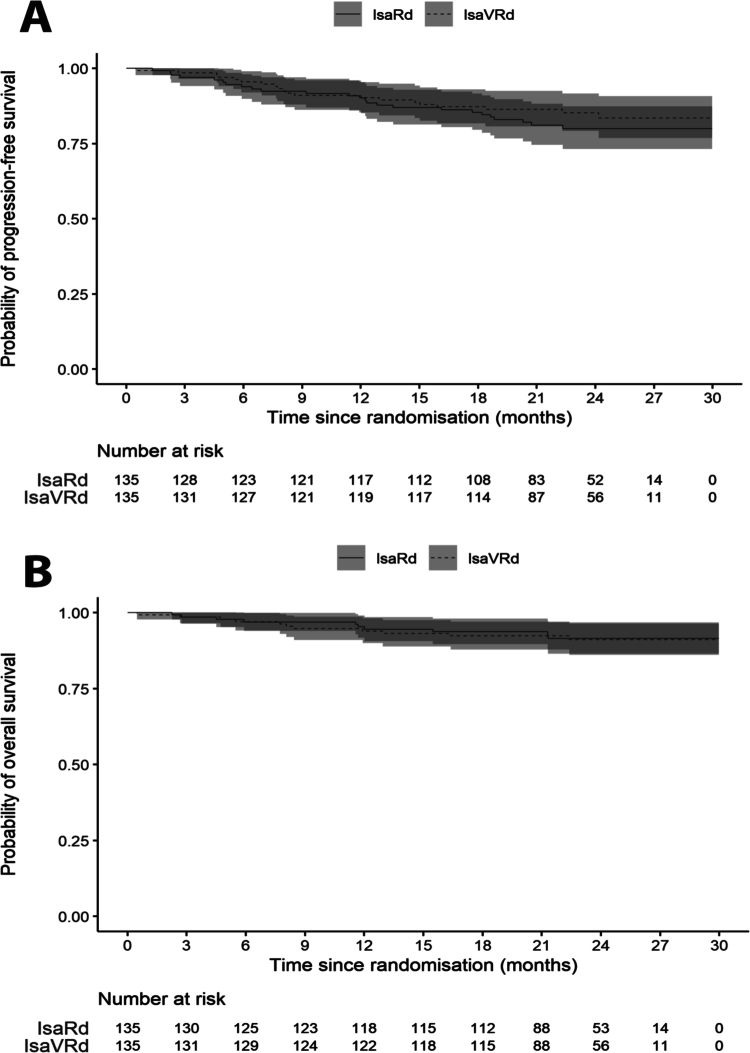
Progression-free survival and overall survival in the ITT population. (**A**) Kaplan–Meier estimates of PFS among patients in the intention-to-treat population. (**B**) Kaplan–Meier estimates of OS among patients in the intention-to-treat population.

With a median follow-up of 23.5 months, PFS and overall survival (OS) data are still immature; 21 progression events and 10 deaths had occurred in the IsaRd arm and 13 and 11 in the Isa-VRd arm, respectively. The causes of death are described in Extended Data Table 2. Estimated 24 months PFS and OS were 80.0% (95% CI 73.3 to 87.4) and 91.5% (95% CI 86.5 to 96.8) for IsaRd, and 85.2% (95% CI 79.2 to 91.7) and 91.1% (95% CI 86.1 to 96.4) for Isa-VRd, respectively (Extended Data Fig. 2a,b).

### Safety

The most common adverse events (AEs) (occurring in ≥10% of patients in either group) are shown in Table 3. Overall, the most common events were neutropenia (61% in IsaRd versus 56% in Isa-VRd), diarrhea (48% in both arms) and infection (39% versus 47%). The thrombocytopenia incidence rate was higher in the Isa-VRd arm compared with the IsaRd arm: 37 (27) and 19 (14) any grades, and 16 (12) and 8 (5) grade 3 and above. There were 164 AEs of grade 2 and above resulting in temporary or permanent discontinuation of bortezomib in the Isa-VRd arm among 79 patients described in Extended Data Table 1. Treatment-emergent AEs leading to death are described in Extended Data Table 2.

**Table 3:** Most common AEs of any grade (worst grade by patient) during treatment in the safety population^a^

Event, no. of patients (%)	IsaRd (*N* = 135)		Isa-VRd (*N* = 135)	
	**Any grade**	**≥Grade 3**	**Any grade**	**≥Grade 3**
**Hematologic AE**				
Neutropenia	82 (61)	61 (45)	77 (57)	53 (40)
Lymphopenia	38 (280)	33 (24)	53 (39)	44 (33)
Anemia	27 (20)	7 (5)	30 (22)	13 (10)
Thrombocytopenia	19 (14)	8 (5)	37 (27)	16 (12)
**Nonhematologic AE**				
	**Any grade**	≥**Grade 2**	**Any grade**	≥**Grade 2**
Diarrhea	65 (48)	30 (22)	66 (49)	39 (29)
Constipation	41 (30)	19 (14)	52 (39)	30 (22)
Nausea	21 (16)	7 (5)	18 (13)	6 (4)
**Infections and infestations**				
Infection of other types	48 (36)	35 (28)	61 (45)	48 (36)
Infection of the respiratory system	64 (47)	54 (40)	65 (48)	47 (35)
Covid-19	59 (44)	31 (23)	55 (41)	34 (24)
Rash	16 (12)	9 (7)	21 (16)	12 (9)
Erythema	—	—	17 (13)	6 (4)
Asthenia	48 (36)	18 (14)	41 (30)	24 (18)
Pain	37 (27)	23 (17)	36 (27)	18 (14)
Muscle spasms	28 (21)	9 (7)	27 (20)	7 (5)
Peripheral Edema	27 (20)	10 (7)	48 (36)	18 (14)
Pyrexia	17 (13)	9 (7)	—	—
Weight decreased	26 (19)	12 (9)	21 (16)	12 (9)
Dyspnea	16 (12)	9 (7)	—	—
Cough	—	—	16 (12)	5 (4)
Insomnia	14 (10)	6 (5)	14 (10)	6 (4)
**Nervous system disorders**				
Peripheral neuropathy	38 (28)	13 (10)	70 (52)	37 (27)
Other	41 (30)	17 (13)	38 (28)	19 (14)
**Psychiatric disorders**	32 (24)	17 (13)	33 (24)	22 (16)
**Eye disorders**	19 (14)	12 (8)	20 (15)	10 (7)
**Hepatobiliary disorders**	19 (14)	13 (9)	—	—
**Renal and urinary disorders**	18 (13)	14 (9)	24 (18)	16 (12)
**Cardiac disorders**	—	—	15 (11)	11 (8)
**Vascular disorders**	34 (25)	23 (17)	36 (27)	21 (15)
**Hypokalemia**	15 (11)	11 (8)	16 (12)	11 (8)

Shown are listed AEs of any grade and ≥grade 3 for hematologic AEs, and any grade and ≥grade 2 for nonhematologic AEs that were reported in at least 10% of patients in either treatment group.

^a^The safety population included all patients who received at least one dose of study treatment.

Solid tumor second primary malignancies (14 events) were reported in 12 patients (6 (4%) in the IsaRd arm and 6 (4%) in the Isa-VRd arm) (Extended Data Table 3).

There were 271 nervous system disorder AEs reported, of which 163 (60%) neuropathy, that developped in 38 (28%) patients in IsaRd and 70 (52%) patients in Isa-VRd arms (Table 3). Peripheral neuropathy ≥grade 2 occurred in 13 (10%) patients (1 patient had grade 3 neuropathy) in the IsaRd arm, and in 37 (27%) patients (4 patients had grade 3 neuropathy) in the Isa-VRd arm. Sixteen (10%) patients discontinued bortezomib related to nervous system disorders ≥grade 2.

## Discussion

At the time of analysis of the primary endpoint of the BENEFIT study, the addition of bortezomib to the IsaRd regimen, significantly improved the MRD 10^−5^ negativity rate at 18 months from randomization. Isa-VRd significantly increased all other MRD endpoints from 12 months, including 10^−6^ MRD negativity rates, the proportion of patients with MRD negativity and ≥CR, and the ≥CR rate at 18 months. The MRD benefit was consistent across subgroups. Because the data are immature regarding survival analysis, there was no difference observed for survival times in the BENEFIT study. However, given the MRD 10^−5^ and 10^−6^ negativity rates observed with Isa-VRd in BENEFIT, we believe these high rates of MRD will potentially translate into prolonged survival times compared with those observed in MAIA. Indeed, MRD is considered a surrogate marker for survival endpoints in NDMM, including in TI populations^16–18^. The BENEFIT results, along with those of IMROZ^19^, confirm that the quadruplet-based isatuximab combined with VRd regimen is a new SOC regimen for the patients with NDMM that are TI.

The MAIA trial (phase 3 DRd versus Rd) reported for the first time on the anti-CD38-targeting immunotherapy-based Rd combination in the NDMM TI population. In the primary analysis of MAIA, the MRD negativity rate was higher in DRd compared with Rd (24% versus 7%; *P* ≤ 0.001)^1^. In MAIA updated reports, DRd led to improved rates of MRD negativity at the 10^−5^ threshold compared with the SOC (28.8% versus 9.2%, *P* < 0.0001)^20^. The DRd and IsaRd data observed in MAIA and BENEFIT studies suggest similar regimens, although daratumumab and isatuximab have different mechanisms of action and epitopes in targeting CD38 (ref. ^21^).

A quadruplet-based CD38-targeting immunotherapy daratumumab-containing regimen has been studied in a NDMM TI population, to improve on MAIA DRd. In ALCYONE (phase 3 daratumumab combined to bortezomib, melphalan and prednisone versus VMP), daratumumab combined to bortezomib, melphalan and prednisone led to improved rates of MRD negativity at the 10^−5^ threshold compared with the SOC (26.9% versus 7.0%, *P* < 0.0001), and at the more stringent threshold of 10^‒6^ (9.1% versus 0.8%, *P* < 0.0001)^20,22^. MAIA remained the best SOC for the NDMM TI population to date when taking into consideration the safety–efficacy balance, and therefore the study of a new quadruplet-based CD38-targeting immunotherapy with VRd became important in the NDMM TI population.

There are limited reports of the MRD negativity rate at the more stringent 10^−6^ threshold, a more difficult MRD negativity rate to improve compared with the 10^−5^ threshold^16^, as demonstrated in MAIA (28.8% and 9.2%, respectively)^20^. We observed an improved MRD negativity rate at 10^−6^ in both arms in BENEFIT at 18 months compared with MAIA. The BENEFIT data support the Isa-VRd regimen as a new quadruplet-based CD38-targeting immunotherapy SOC for NDMM TI patients aged 65 to 79 years.

The MRD negativity rates in studies for patients with NDMM that are transplant-eligible (TE) remain higher overall than the one reported in BENEFIT^23–26^. The PERSEUS trial (phase 3 DVRd versus VRd)^23^, in the context of patients with multiple myeloma that are TE, reported higher MRD negativity rates at the 10^−6^ threshold in DVRd compared with VRd (65.1% versus 32.2%, *P* < 0.0001). This suggests that autologous transplantation remains the SOC for NDMM patients who could be candidates for the intensification. However, the benefit of the transplantation has been clearly demonstrated only in patients up to 65 years old^23^, and to a lesser extent in patients ≥65 years old^27^. The combined results from IMROZ^19^ and BENEFIT suggest we rethink the age limit cutoff for consideration of a patient that is TE, given the benefit–risk balance in favor of the quadruplet regimen Isa-VRd over the autologous transplantation.

There are differences across the BENEFIT and IMROZ studies on the cumulative dose density of the proteasome inhibitor bortezomib. Bortezomib was given on a twice-weekly schedule in IMROZ (overall 24 twice-weekly infusions over a 6-month period) and as a less-dense schedule but over a more prolonged time in BENEFIT (overall 48 weekly infusions over 18 months). It will be of importance to determine the best schedule for patients to mitigate side effects, notably peripheral neuropathy, and also verify whether the weekly less-dense schedule of bortezomib is similarly active.

Prespecified subgroup analyses showed consistent MRD improvement with Isa-VRd versus IsaRd across clinically relevant subgroups, including patients with International Staging System (ISS) stage III disease or high-risk cytogenetics. The Isa-VRd with weekly bortezomib administration benefited all patients aged 65 to 79 years, including those who might have presented over time with frailty or comorbid conditions.

There are limitations in our BENEFIT study, including restricting the upper age for recruitment to 79 years, the use of intravenous isatuximab, the prolonged use of bortezomib up to 18 months and the treatment schema given as isatuximab and lenalidomide until progression. It would be of interest to study a Isa-VRd regimen in patients over the age of 79 years, characterized by frail conditions. The phase 2 REST study (NCT04939844) from the European Nordic group is studying the Isa-VR no dexamethasone in NDMM with frail conditions. It is possible that a triplet-based regimen with isatuximab and lenalidomide combined with a very careful administration schema of bortezomib, and the absence of corticoids, may substantially improve survival in patients with frail conditions, allowing only one line of treatment and a planned fixed duration of treatment in the future. A study of the subcutaneous formulation of isatuximab is ongoing in TI and TE patients upfront (ISASOCUT NCT05889221and GMMG-HD8 NCT05804032studies).

Finally, the Isa-VRd regimen is simplified after 18 months from randomization, and the isatuximab and lenalidomide regimen is pursued in both arms until progression. A fixed-duration isatuximab and lenalidomide regimen would certainly improve the safety profile, particularly in the long run, maybe even long-term side effects such as second primary malignancies, and improve the patients’ quality of life. Given the absence of data suggesting that a regimen with fixed duration could provide the same efficacy results as current treatment regimens given continuously, it was difficult to plan this option in BENEFIT at the study start. We could amend the protocol if such data were reported. The Intergroupe Francophone of Myeloma (IFM) CONFIRM phase 3 study (study comparing continuous versus fixed-duration therapy with daratumumab, lenalidomide and dexamethasone for relapsed multiple myeloma, NCT03836014) may help to provide these data.

The safety profile of both the IsaRd and Isa-VRd arms was consistent with the known safety profiles for DRd, and additionally, treatment discontinuations and deaths because of AEs in the Isa-VRd arm were consistent with the IsaRd-treated population.

In summary, the results from the BENEFIT study demonstrated a meaningful benefit of the quadruplet-based isatuximab plus VRd regimen compared with IsaRd. These data support Isa-VRd as a new SOC for patients with NDMM that are TI and aged 65 to 79 years over the current triplet-based SOC DRd.

## Methods

### Trial design and oversight

This open-label, multicenter parallel arms phase 3 trial randomly assigned patients between 7 September 2021 and 2 September 2022, recruited across 60 centers in France. Patients were randomly (1:1 ratio) assigned to Isa-VRd or IsaRd until progression, one cycle being 28 days long. Randomization was stratified by age (<75 and ≥75 years), cytogenetic risk at baseline as assessed by fluorescence in situ hybridization (Supplementary Appendix) and type of center (based on volume and teaching status). There was no selection of patients.

### Inclusion and ethics

The study was sponsored by Centre Hospitalier Universitaire (CHU) Poitiers, France, in collaboration with the IFM. The IFM and CHU Poitiers in collaboration with the investigators, designed the trial and compiled and maintained the data collected by the investigators. All authors had access to the data and were not restricted by confidentiality agreements. All authors reviewed, revised and approved the manuscript. The sponsor and authors vouch for data accuracy and completeness and for adherence to the study protocol.

An independent ethics committee (CPP Est-II, Besancon, France, eudra CT 2020-004602-59) approved the study protocol along with agence nationale de securite du medicament (ANSM). The study was conducted in accordance with the International Conference on Harmonization Good Clinical Practice guidelines, the principles originating from the Declaration of Helsinki. All patients provided written informed consent.

### Patients

Eligible patients had NDMM TI and were aged 65 to 79 years (Supplementary Appendix). Sex was collected and reported in the trial.

### Inclusion and exclusion criteria

Key inclusion criteria included:

1. Must be able to understand and voluntarily sign an informed consent form
2. Must be able to adhere to the study visit schedule and other protocol requirements
3. Life expectancy of >6 months
4. Subject, male or female, must be at least ≥65 years of age and <80 years of age
5. Must have a newly diagnosed multiple myeloma requiring therapy (SLiM CRAB criteria)
  Monoclonal plasma cells in the bone marrow ≥10% or presence of a biopsy proven plasmacytoma. Clonality should be established by showing κ/λ-light-chain restriction on flow cytometry, immunohistochemistry or immunofluorescence. Bone marrow plasma cell percentage should preferably be estimated from a core biopsy specimen; in case of a disparity between the aspirate and core biopsy, the highest value should be used and any one or more of the following myeloma-defining events:
  Revised International Myeloma Working Group diagnostic criteria for multiple myeloma myeloma-defining events:

- Evidence of end organ damage that can be attributed to the underlying plasma cell proliferative disorder, specifically:
  - Hypercalcemia—serum calcium >0.25 mmol l^−1^ (>1 mg dl^−1^) higher than the upper limit of normal or >2.75 mmol l^−1^ (>11 mg dl^−1^)
  - Renal insufficiency—creatinine clearance ≤40 ml min^−1^ or serum creatinine ≥177 μmol l^−1^ (≥2 mg dl^−1^); measured or estimated by validated equations

- Anemia—hemoglobin value of ≥20 g l^−1^ below the lower limit of normal, or hemoglobin value ≤100 g l^−1^
- Bone lesions—one or more osteolytic lesions on skeletal radiography, computed tomography or positron emission tomography–computed tomography. If bone marrow has <10% clonal plasma cells, more than one bone lesion is required to distinguish from solitary plasmacytoma with minimal marrow involvement
- Any one or more of the following biomarkers of malignancy: clonal bone marrow plasma cell percentage ≥60%, involved/uninvolved serum-free light chain ratio ≥100 (these values are based on the serum Freelite assay (The Binding Site Group, Birmingham, UK)). The involved free light chain must be ≥100 mg l^−1^. One focal lesion on MRI studies (each focal lesion must be 5 mm or more in size)

(6) Must have measurable disease as defined by any of the following:

- IgG myeloma—serum monoclonal paraprotein (M-protein) level ≥1.0 g dl^−1^ or urine M-protein level ≥200 mg per 24 h; or
- IgA, IgM, IgD or IgE multiple myeloma—serum M-protein level ≥0.5 g dl^−1^ or urine M-protein level ≥200 mg per 24 h; or
- light chain multiple myeloma—serum immunoglobulin free light chain ≥10 mg dl^−1^ and abnormal serum immunoglobulin kappa lambda free light chain ratio (only measurable with Freelite by binding site)

(7) Must be nontransplant-eligible nonfrail:

- newly diagnosed and not considered candidate for high-dose chemotherapy with stem cell transplantation
- subject must have a frailty score <2

(8) Eastern Cooperative Oncology Group (ECOG) performance status score of 0, 1 or 2
(9) Adequate bone marrow function, documented within 72 h and without transfusion 72 h before the first intake of investigational product (C1J1) with no growth factor support (1 week), defined as: absolute neutrophils ≥1 × 10^9^ per l, untransfused platelet count ≥75 × 10^9^ per l, hemoglobin ≥8.5 g dl^−1^
(10) Adequate organ function defined as: serum total bilirubin <2× upper limit of normal, creatinine clearance ≥30 ml min^−1^, serum SGOT/AST or SGPT/ALT <3× upper limit of normal
(11) Subjects affiliated with an appropriate social security system
(12) A man who is sexually active with a pregnant woman or a woman of childbearing potential must agree to use a barrier method of birth control, for example a condom with spermicidal foam/gel/film/cream/suppository during the study and for at least 5 months after the last dose of treatment, even he has had a vasectomy. A woman of childbearing potential is any sexually mature female who: (1) has achieved menarche at some point; (2) has not undergone a hysterectomy or bilateral oophorectomy; or (3) has not been naturally postmenopausal (not having menstrual cycles because of cancer therapy does not rule out childbearing potential) for at least 24 consecutive months
(13) A female participant is eligible to participate if she is not pregnant, not breastfeeding and at least one of the following conditions applies: not a woman of childbearing potential or is a woman of childbearing potential who must have a negative serum or urine pregnancy test with a sensitivity of at least 25 mIU ml^−1^ within 10–14 days before and again within 24 h before starting study medication and before each cycle of study treatment. A woman of childbearing potential must understand and agree to continue abstinence from heterosexual intercourse or to use two reliable effective methods of contraception (a very effective method and an effective additional method) simultaneously without interruption: for at least 28 days before starting experimental treatments, throughout the entire duration of experimental treatments, during dose interruptions and for at least 5 months after the last dose of experimental treatments
(14) All patients must understand and accept to comply with the conditions of the lenalidomide pregnancy prevention plan.

Key exclusion criteria included:

1. Subject has a diagnosis of primary amyloidosis, monoclonal gammopathy of undetermined significance or smoldering multiple myeloma. Monoclonal gammopathy of undetermined significance is defined by the presence of serum M-protein <3 g dl^−1^; absence of lytic bone lesions, anemia, hypercalcemia and renal insufficiency related to the M-protein; and (if determined) proportion of plasma cells in the bone marrow of 10% or less^31^. Smoldering multiple myeloma is defined as asymptomatic multiple myeloma with absence of related organ or tissue impairment end organ damage^31^.
2. Subject has a diagnosis of Waldenström’s disease, or other conditions in which IgM M-protein is present in the absence of a clonal plasma cell infiltration with lytic bone lesions.
3. Subject has previous or current systemic therapy or stem cell transplantation for multiple myeloma, with the exception of an emergency use of a short course (equivalent of dexamethasone 40 mg d^−1^ for a maximum 4 days) of corticosteroids before treatment.
4. Subject has a history of malignancy (other than multiple myeloma) within 3 years before the date of randomization (exceptions are squamous and basal cell carcinomas of the skin and carcinoma in situ of the cervix, or malignancy that in the opinion of the investigator, with concurrence with the coordinator investigator, is considered cured with minimal risk of recurrence within 3 years).
5. Subject has had radiation therapy within 7 days of randomization.
6. Subject has had plasmapheresis within 7 days of randomization.
7. Subject is exhibiting clinical signs of meningeal involvement of multiple myeloma.
8. Subject known to be seropositive for history of human immunodeficiency virus or to have hepatitis A active infection.
9. Subject known to have hepatitis B (HBV) active or uncontrolled infection (positive Hepatitis B virus surface antigen (HBsAg) and/or HBV DNA). Patient can be eligible if anti-HBc IgG positive (with or without positive anti-HBs) but HBsAg and HBV DNA are negative. If anti-HBV therapy in relation with previous infection was started before initiation of investigational medicinal product, the anti-HBV therapy and monitoring should continue throughout the study treatment period. Patients with negative HBsAg and positive HBV DNA observed during the screening period will be evaluated by a specialist for start of antiviral treatment: study treatment could be proposed if HBV DNA becomes negative and all the other study criteria are still met.
10. Subject known to have hepatitis C (HCV) active infection (positive HCV RNA and negative anti-HCV). Patients with antiviral therapy for HCV started before initiation of investigational medicinal product and positive HCV antibodies are eligible. The antiviral therapy for HCV should continue throughout the treatment period until seroconversion. Patients with positive anti-HCV and undetectable HCV RNA without antiviral therapy for HCV are eligible.
11. Subject has any clinically important medical or psychiatric condition or disease (for example, uncontrolled diabetes, acute diffuse infiltrative pulmonary disease) in the investigator’s opinion, would expose the patient to excessive risk or may interfere with compliance or interpretation of the study results.
12. Subject has active systemic infection and severe infections requiring treatment with a parenteral administration of antibiotics.
13. Subject has clinically important cardiac disease, including: myocardial infarction within 6 months before randomization, or an unstable or uncontrolled disease/condition related to or affecting cardiac function (for example, unstable angina, congestive heart failure, New York Heart Association Class III–IV), uncontrolled cardiac arrhythmia (National Cancer Institute Common Terminology Criteria for Adverse Events Version 5 grade ≥2) or clinically significant electrocardiogram abnormalities.
14. Subject has known allergies, hypersensitivity or intolerance to steroids, mannitol, pregelatinized starch, sodium stearyl fumarate, histidine (as base and hydrochloride salt), arginine hydrochloride, poloxamer 188, sucrose or any of the other components of study intervention that are not amenable to premedication with steroids and H2 blockers or would prohibit further treatment with these agents, monoclonal antibodies or human proteins, or their excipients (refer to respective package inserts or investigator’s brochure).
15. Subject has known hypersensitivity, allergy to one of the study product (isatuximab, lenalidomide, bortezomib), dexamethasone or to one of the excipients.
16. Subject has acute diffuse infiltrative pneumopathy, pericardial disease.
17. Subject has plasma cell leukemia (according to World Health Organization criterion: ≥20% of cells in the peripheral blood with an absolute plasma cell count of more than 2 × 10^9^ per l) or POEMS syndrome (polyneuropathy, organomegaly, endocrinopathy, monoclonal protein and skin changes).
18. Subject is known or suspected of not being able to comply with the study protocol (for example, because of alcoholism, drug dependency or psychological disorder). Subject has any condition for which, in the opinion of the investigator, participation would not be in the best interest of the subject (for example, compromise their well-being) or that could prevent, limit or confound the protocol-specified assessments. Subject is taking any prohibited medications.
19. Subject has had major surgery within 2 weeks before randomization or has not fully recovered from surgery, kyphoplasty or vertebroplasty is not considered major surgery.
20. Subject has received an investigational drug (including investigational vaccines) within 14 days or five half-lives of the investigational drug before initiation of study intervention, whichever is longer, or used an invasive investigational medical device within 4 weeks before randomization or is currently enrolled in an interventional investigational study. In case of very aggressive disease (acute leukemia) delay could be shortened after agreement between sponsor and investigator, in absence of residual toxicities from previous therapy.
21. Subject refuses to consent or is protected by legal regime (under judicial protection, guardianship, trusteeship).
22. Subject has contraindications to required prophylaxis for deep vein thrombosis and pulmonary embolism.
23. Incidence of gastrointestinal disease that may importantly alter the absorption of oral drugs.

### Trial treatments

All patients received isatuximab + lenalidomide + dexamethasone and/or bortezomib from cycle 1 to cycle 12, followed by isatuximab + lenalidomide and/or bortezomib from cycle 13 to cycle 18, and isatuximab + lenalidomide from cycle 19 to progression (Supplementary Appendix). Bortezomib was permanently interrupted at cycle 18. Isatuximab was given at a dose of 10 mg kg^−1^ administered intravenously, every week at days 1, 8, 15 and 22 for the first cycle, then every other week at days 1 and 15 from cycle 2 onward, and once monthly (day 1) from cycle 13 to progression. Lenalidomide was given orally at 25 mg daily on days 1–21 from cycle 1 up to progression. Dexamethasone was given orally at 20 mg weekly at days 1, 8, 15 and 22 until cycle 12, then was permanently stopped. In the bortezomib arm, bortezomib was given at 1.3 mg per m^2^, weekly at days 1, 8 and 15, subcutaneously from cycle 1 to 12, and bimonthly at days 1 and 15 from cycle 13 to 18 (Supplementary Fig. 1).

### Endpoints and assessments

The primary endpoint was MRD rate at or below a sensitivity threshold of 10^−^^5^ at 18 months from randomization. The key secondary endpoints for response evaluation, MRD assessments, survival and safety are detailed in the Supplementary Appendix.

MRD was performed on bone marrow aspiration in patients who achieved at least ≥PR for the primary endpoint timepoint of 18 months. The patients with primary refractory disease, stable disease and minor response, along with patients failing or not tested for MRD analysis, were considered as patients with positive MRD at 10^−5^. The MRD test was centrally and primarily determined by next-generation sequencing (NGS) with a 10^−6^ sensitivity (A. Loiseau and J. Corre, Toulouse Oncopole, France). In the case of failure to perform MRD by NGS, MRD assessment was then performed centrally using multiparametric flow cytometry with a 10^−5^ sensitivity (F. Vergez, Toulouse Oncopole, France) (Supplementary Appendix).

### Statistical analysis

Assuming that 15% of patients would be MRD negative at 18 months in the IsaRd arm (based on approximated initial results from MAIA), inclusion of 242 patients would give an 80% power to detect an improvement from 15% to 30% in the Isa-VRd arm at a two-sided alpha level of 0.05. To account for potential dropouts, the plan was to enroll 270 patients.

The primary analysis was performed in the intention-to-treat (ITT) population, which included all randomized patients. The safety population included all patients who had received at least one dose of the assigned treatment. The primary endpoint was compared between treatment groups using a Wald test and treatment effect was assessed by OR and 95% CI using a mixed logistic regression with treatment as the explanatory variable and adjusting for randomization stratification factors. All other binary secondary endpoints were analyzed similarly to the primary endpoint.

For time-to-event endpoints, distribution was estimated and plotted using the Kaplan–Meier method (PFS, OS) or Gray cumulative incidence method in case of competition (time to first response, time to best response). Endpoints were compared between arm, and treatment effect was assessed by HR (or cause-specific HR in case of competing risks) and 95% CI using a Cox proportional hazard model with treatment as the explanatory variable and adjusting for randomization stratification factors. When data were not mature enough, no test was performed and no HR was estimated.

Homogeneity of treatment effect on the primary endpoint was checked by plotting effect in predefined subgroups using a Forest plot and testing for significance of an interaction term included in a logistic regression model.

No interim analysis was planned and all endpoints were tested at a two-sided alpha level of 5% without correction for multiplicity. All statistical analyses were performed using R software v.4.2.1.

### Reporting summary

Further information on research design is available in the Nature Portfolio Reporting Summary linked to this article.

## Online content

Any methods, additional references, Nature Portfolio reporting summaries, source data, extended data, supplementary information, acknowledgements, peer review information; details of author contributions and competing interests; and statements of data and code availability are available at 10.1038/s41591-024-03050-2.

### Supplementary information

Supplementary Information

Reporting Summary

## Figures and Tables

**Extended Data Table 1:**
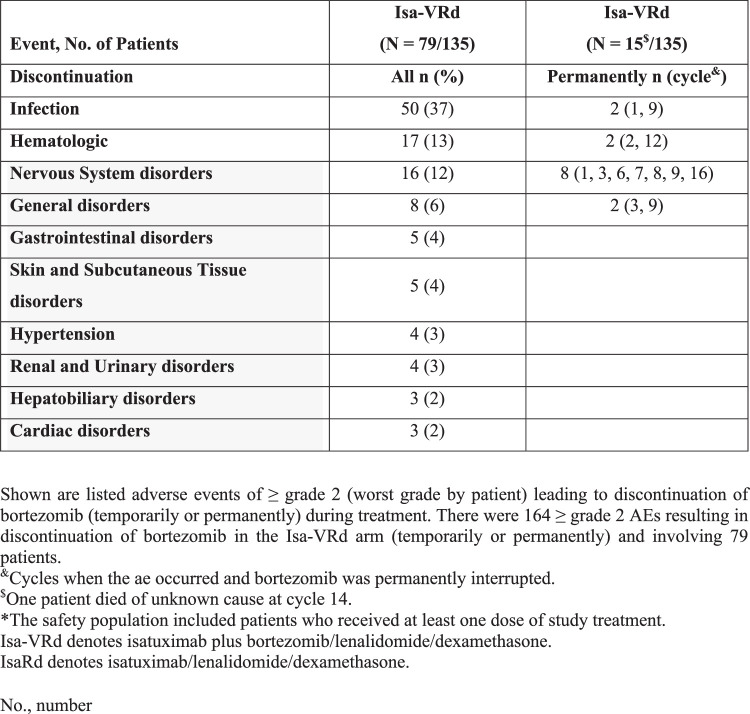
Overview of the Adverse Events of ≥ Grade 2 (worst grade by patient) leading to discontinuation of Bortezomib (temporarily or permanently) during Treatment in the Safety Population*

**Extended Data Table 2:**
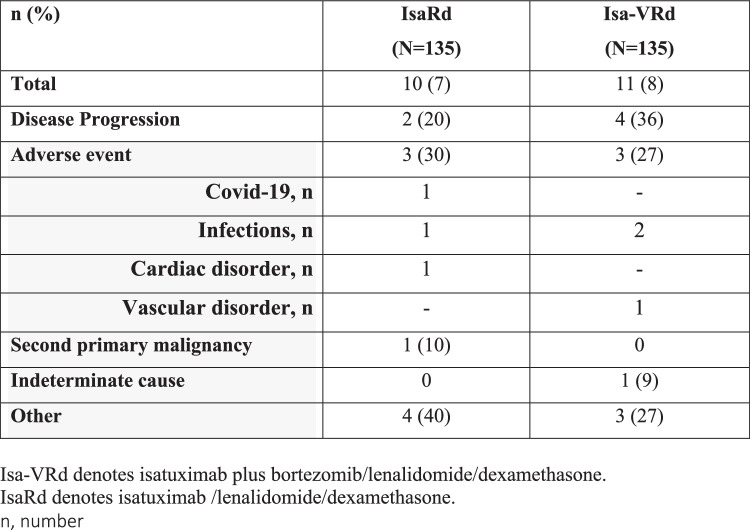
Cause of death in the BENEFIT study

**Extended Data Table 3:**
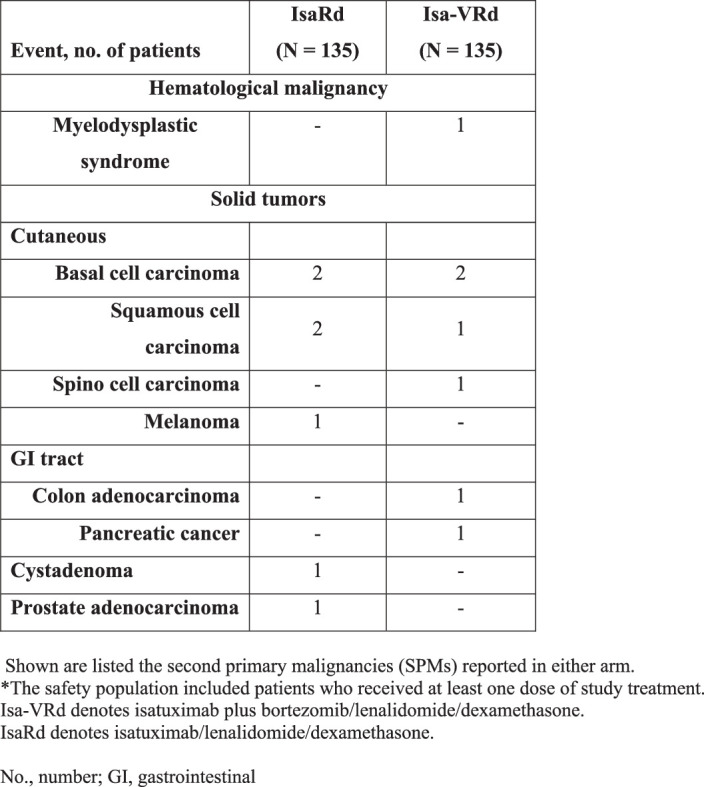
Overview of second primary malignancies (SPMs) in the Safety Population*

## Data Availability

Data supporting this article are part of an ongoing clinical trial and are not publicly available. Data will be considered for sharing once the product and indication has been approved by major health authorities (for example, the US Food & Drug Administration, the European Medicines Agency), with restriction due to data privacy regulations, and the informed consent. Requests for de-identified patient data by researchers with proposed use of the data can be made to the corresponding author with specific data needs, analysis plans and dissemination plans. Those requests will be reviewed by a study steering committee (IFM group) and the study sponsor for release upon publication. Response will typically be given in 3 months. The trial protocol and statistical analysis plan can be found in the Supplementary Information.

## Code availability

The IFM eCRF use the V8.2.30 Ennov version. All statistical analyses were performed using R software v.4.2.1.
