# Supplementary material for: Isatuximab, lenalidomide, dexamethasone and bortezomib in transplant-ineligible multiple myeloma: the randomized phase 3 BENEFIT trial
Source: Nat Med. 2024 Jun 3;30(8):2235–41. doi: 10.1038/s41591-024-03050-2 (PMC11333283; doi:10.1038/s41591-024-03050-2)
Supplement: Supplementary file 1 — Supplementary Information [file 41591_2024_3050_MOESM1_ESM.pdf]

nature medicine

Supplementary information https://doi.org/10.1038/s41591-024-03050-2

# Isatuximab, lenalidomide, dexamethasone and bortezomib in transplant-ineligible multiple myeloma: the randomized phase 3 BENEFIT trial

In the format provided by the authors and unedited

**Supplementary Appendix**

This appendix has been provided by the authors to give readers additional information about their work.

**Supplement to:** Leleu X and Hulin C et al. Isatuximab plus lenalidomide and dexamethasone with light bortezomib versus isatuximab plus lenalidomide and dexamethasone in newly diagnosed transplant ineligible Multiple Myeloma. The BENEFIT (IFM 2020-05) study.

**Table of Contents**


**Collaborators.** ..... 3

**Study Sites.** ..... 4

**Supplementary Methods.** ..... 6

**Patients** ..... 6

**Pre-injection Medications** ..... 11

**Endpoints and Assessments** ..... 11

**MRD assessment and method.** ..... 12

**The secondary objectives** ..... 13

**Cytogenetic risk assessment.** ..... 15

**Data and Safety Monitoring Board (DSMB).** ..... 18

**Supplemental appendix Figure 1. Trial Design.** ..... 19

**References** ..... 20

**Collaborators.**

The following principal investigators participated in the BENEFIT study

Perrot Aurore[1], Leleu Xavier[2], Jaccard Arnaud [3], Montes Lydia[4], Moralleau Jean-Pierre[4], Karlin Lionel[5], Manier Salomon[6], Godmer Pascal[7], Chalopin Thomas[8], Slama Borhane[9], Bastie Jean-Noel[10], Caillot Denis[10], Laribi Kamel[11], Hulin Cyrille[12], Dingremont Claire[13], Roul Christophe[14], Mariette Clara[15], Rigaudeau Sophie[16], Calmettes Claire[17], Dib Mamoun[18], Tiab Mourad[19], Vincent Laure[20], Delaunay Jacques[21], Santagostino Alberto[22], Macro Margaret[23], Bourgeois Emmanuelle[24], Touzeau Cyrille[25], Mohty Mohamad[26], Orsini Piocelle Frederique[27], Gay Julie [28], Araujo Carla[28], Bareau Benoit[29], Lebreton Pierre[30], Zarnitsky Charles[30], Tabrizi Reza[31], Rascalou Waultier Agathe[32], Jourdan Eric[32], Frenzel Laurent[33], Le Calloch Ronan[34], Chalayer Emilie[35], Collet Philippe[35], Braun Thorsten[36], Lachenal Florence[37], Corm Selim[38], Kennel Celine[39], Belkhir Rakiba[40], Blade Jean-Sebastien[41], Joly Bertrand[42], Demarquette Helene[43], Robu-Cretu Daniela[44], Newinger-Porte Muriel[45], Richez-Olivier Valentine[46], Benbrahim Omar[47], Garderet Laurent[48] , Decaux Olivier[49], Allangba Olivier[50], De Thomas De Labarthe Adrienne[51], Rey Philippe[52], Schiano De Colella Jean-Marc[53], Stoppa Anne-Marie[53], Dorvaux Veronique[54], Vignon Marguerite[55], Roland Virginie[56], Salmeron Geraldine[57], Godet Sophie[58], Garidi Reda[59], Maloisel Frederic[60], Le Ray Emmanuelle[61], El Yamani Abderrazak[62], Fitoussi Olivier[63], Orfeuvre Hubert[64], Kaphan Regis[65], Belhadj Karim[66], Royer Bruno[67], Thannberger Alexia[68]

The following clinical research coordinators participated in the BENEFIT study

Bentaberryrosa Alexandre[1], Pansard Myriam[2], Bompart Frederica[3], Aleme Audrey[4], Abhamon Maelenn[5], Liberal Olivier[6], Le Brun Chloe[7], Gaborit Vincent[8], Nasri Safa[9], Devaux Laetitia[10], Mimita Sameh[11], Kadi-Hanifi Mahmoud[12], Latry Vanessa[13], Biais-Sauvetre Fanny[14], Dodge Meryl[15], Cavelot Sebastien[16], Gibiat Stephanie[17], Denous-Chataigner Paul[18], Antonio Marie[19], Damiano Alessandra[20], Hugon Nathalie[21], Houyou Dyhia[22], Abonnet Veronique[23], Gonda Benoite[24], Garnier Heloïse[25], Haidara Aminata[26], Durand Carole[27], Barreau Angeline[28], Huet Sylvie[29], Gibaux Audrey[30], Dolbeault Melanie[31], Torres Laetitia[32], Bouhaouche Rym[33], Jolas Stephanie[34], Durieux Coralie[35], Edouart Geoffrey[36], Mollon Alexandra[37] , Habet Tarik[38], Desbrosses Claudine[39], Mamoune Asmaa[40], Dachary Corinne[41], Matray Marie[42], El Azouzi Paquez Virginie[43], Declercq Herve[44], Haby Celine[45], Hamadi Sarah[46], Michel Olivier[47], Chellouf Rimal[48], Martin Amelie[49], Lemoine Matthieu[50], Ruiz Gabrielle[51], Duby Lucie[52], Quang Mathieu[53], Fery Catherine[54], Ben M'barek Rim[55], Touhami Fatima[56], Diouf Daba[57], Bondon Florine[58], Moreau Léa[59], Guillot Isabelle[60], Kadiri Ouafae[62], Vincent Muriel[63], Chagros Melanie[64], Doux Nathalie[65], Dellah Mounira[67], Le Breton Aude[68]

**Study Sites.**

The following study sites enrolled at least 1 patient in the BENEFIT study:

[1] University Hospital of Toulouse, IUCT Oncopole, Toulouse

2 Service d'hématologie, CIC 1082, U1313, CHU, University, Poitiers

3 Hematologie, CHU, Limoges

[4] Hematology, CHU, Amiens

[5] Hôpital Lyon Sud, Pierre-Benite

[6] Hematology, University Hospital, Inserm U-S1277 and CNRS UMR9020, Lille

[7] CH Bretagne Atlantique, Vannes

[8] Department of Hematology, CHU, Tours

[9] Hospital center, Avignon

[10] Hematology, University Hospital, Inserm U1231, University of Burgundy Franche-Comté Dijon

[11] Department of Hematology, CH, Le Mans

[12] CHU, Bordeaux

[13] Department of Internal Medicine, Tarbes-Lourdes Hospital, Tarbes

[14] CH La Rochelle

[15] Hematology, CHU, Grenoble

[16] CH, Versailles, Service d'Hématologie – Le Chesnay

[17] CH, Périgueux

[18] University Hospital, Angers

[19] Hematology, CH Departemental de La Roche-sur-Yon

[20] CHU, Montpellier

[21] Hôpital privé du confluent 2, Nantes

[22] Department of hematology, CH, Troyes

[23] IHBN – CHU. Hematology, CHU, Caen

[24] GHICL, Lille

[25] CHU, Nantes

[26] Sorbonne University, Saint-Antoine Hôpital (AP-HP), Hematology, UMRs 938, Paris

[27] Hématologie, CH, Annecy Genevois -

[28] Hematology, CH de la côte basque

[29] Hematology, Les Hôpitaux Privés Rennais Cesson Sévigné - Vivalto Santé, Brittany

[30] Hematology, CH, Le Havre

[31] Hematology, CHI de Mont De Marsan

[32] Hematology, CHU, Nîmes

[33] Hematology, Necker Hospital, Paris

[34] Hematology, CH de Cornouaille, Quimper Concarneau

[35] Hematology, CHU, Saint-Etienne

[36] Hematology, Avicenne hospital APHP, Bobigny
[37] CH Pierre Oudot, Bourgoin-Jallieu
[38] Medipole de Savoie, Challes les Eaux
[39] CH William Morey, Chalon sur Saône
[40] Rheumatology, Hopital Bicetre, AP-HP, Universite Paris Saclay, Paris
[41] Onco-Hematology Department, HIA Sainte Anne, Toulon
[42] CH Sud Francilien, Corbeil Essonnes
[43] Hematology, CH, Dunkerque
[44] Hematology, CH, Lens
[45] Hôpital Emile Muller, Mulhouse
[46] CHU de Nice
[47] Hematology, CHU, Orléans
[48] Hopital Pitié Salpetriere, hématologie, APHP, Paris
[49] Hematology, UMR U1236 InsermUniversity Hospital, Rennes
[50] CH Yves Le Foll, Hématologie-Oncologie, Saint Brieuc
[51] Hematology, CH Louis Pasteur, Le Coudray
[52] Centre de Lutte Contre le Cancer Léon Bérard, hématologie.
[53] Institut Paoli Calmettes, Marseille
[54] Hematology, CH Régional Metz-Thionville, Metz
[55] Hematology, Cochin Hospital, AP-HP, Centre Paris-Cité University, Paris
[56] CH, Perpignan
[57] CHI Poissy-Saint-Germain-en-laye
[58] Hôpital Robert Debré,
[59] Hematology, Hospital Saint Quentin.
[60] Hematology, Oncologie Libérale, Clinique St Anne, Strasbourg
[61] CH, Argenteuil
[62] CH, Simone Veil de Blois
[63] Bordeaux Polyclinique Nord Aquitaine
[64] CH, Bourg-en-Bresse - Hopital de Fleyriat
[65] CH, Cannes
[66] C.H.U. Henri Mondor
[67] Hôpital Saint-Louis, Paris
[68] Hematology, CH, Saint Malo

**Supplementary Methods.**

Please see the full BENEFIT protocol for additional methodology details.

**Patients**

Inclusion criteria. Eligible patients

1. Must be able to understand and voluntarily sign an informed consent form
2. Must be able to adhere to the study visit schedule and other protocol requirements
3. Life expectancy > 6 months
4. Subject, male or female, must be at least ≥ 65 years of age and < 80 years of age
5. Must have a Newly diagnosed Multiple Myeloma requiring therapy (SLiM CRAB criteria) *(see appendix 18.2)*

5.1. Monoclonal plasma cells in the bone marrow ≥10% or presence of a biopsy proven plasmacytoma*

**Clonality should be established by showing κ/λ-light-chain restriction on flow cytometry, immunohistochemistry, or immunofluorescence. Bone marrow plasma cell percentage should preferably be estimated from a core biopsy specimen; in case of a disparity between the aspirate and core biopsy, the highest value should be used and any one or more of the following myeloma defining events*:

5.2.Revised International Myeloma Working Group diagnostic criteria for multiple myeloma

Myeloma defining events:

- Evidence of end organ damage that can be attributed to the underlying plasma cell proliferative disorder, specifically:
  - Hypercalcemia: serum calcium >0.25 mmol/L (>1 mg/dL) higher than the upper limit of normal or >2.75 mmol/L (>11 mg/dL)
  - Renal insufficiency: creatinine clearance ≤40 mL per min[†] or serum creatinine ≥177 µmol/L (≥2 mg/dL)

  [†]*Measured or estimated by validated equations*
- Anemia: hemoglobin value of ≥ 20 g/L below the lower limit of normal, or hemoglobin value ≤100 g/L
- Bone lesions: one or more osteolytic lesions on skeletal radiography, CT, or PET-CT [‡]

  [‡]*If bone marrow has less than 10% clonal plasma cells, more than one bone lesion is required to distinguish from solitary plasmacytoma with minimal marrow involvement*
- Any one or more of the following biomarkers of malignancy:
  - Clonal bone marrow plasma cell percentage* ≥60%
  - Involved/uninvolved serum free light chain ratio ≥100[§]

  [§]*These values are based on the serum Freelite assay (The Binding Site Group, Birmingham, UK).*

*The involved free light chain must be ≥100 mg/L.*

- >1 focal lesion on MRI studies (*Each focal lesion must be 5 mm or more in size.)*

6. Must have measurable disease as defined by any of the following:
   IgG myeloma: Serum monoclonal paraprotein (M-protein) level ≥1.0 g/dL or urine M- protein level ≥200 mg/24 hours;
   or
   IgA, IgM, IgD, or IgE multiple myeloma: serum M-protein level ≥0.5 g/dL or urine M- protein level ≥200 mg/24 hours;
   or
   Light chain multiple myeloma: urine M- protein level ≥200 mg/24 hours or if not quantifiable in urines: Serum immunoglobulin free light chain ≥10 mg/dL and abnormal serum immunoglobulin kappa lambda free light chain ratio (measurable with freelite® by Binding site)*
   * Another laboratory method can be used. In such a case, the same method must be used along the study for a given patient.
7. Must be nontransplant eligible and not frail

7.1. Newly diagnosed and not considered candidate for high-dose chemotherapy with SCT.

7.2. Subject must be not frail.

8. Eastern Cooperative Oncology Group (ECOG) performance status score of 0, 1, or 2 *(see appendix 18.3)*.
9. Adequate bone marrow function, documented within 72 hours and without transfusion 72 hours prior to the first intake of investigational product (C1J1) with no growth factor support (one week), defined as:
   - Absolute neutrophils ≥ 1 $x10^9$/L,
   - Untransfused Platelet count ≥ 75 $x10^9$/L,
   - Hemoglobin ≥8.5 g/dL.
10. Adequate organ function documented within one week prior to the first intake of investigational product (C1J1) defined as:
    - Serum total bilirubin < 2x upper limit of normal (ULN),
    - Creatinine clearance ≥ 30ml/min calculated with MDRD formula,
    - Serum SGOT/AST or SGPT/ALT < 3x upper limit of normal (ULN).
11. Subjects affiliated with an appropriate social security system.
12. A man who is sexually active with a pregnant woman or a woman of childbearing potential must agree to use a barrier method of birth control e.g., condom with spermicidal foam/gel/film/cream/suppository during the study and for at least 5 months after the last dose of

treatment, even he has had a vasectomy.

13. A female participant is eligible to participate if she is not pregnant, not breastfeeding, and at least one of the following conditions applies:
    13.1. Not a female of childbearing potential
    Or
    13.2. A FCBP* who must have a negative serum or urine pregnancy test with a sensitivity of at least 25 mIU/mL within 10 – 14 days prior to and again within 24 hours prior to starting study medication and before each cycle of study treatment.
    A FCBP* must understand and agree to continue abstinence from heterosexual intercourse or to use 2 reliable effective methods of contraception (a very effective method and an effective additional method) simultaneously without interruption:
    13.2.1. For at least 28 days before starting experimental treatments,
    13.2.2. Throughout the entire duration of experimental treatments,
    13.2.3. During dose interruptions,
    13.2.4. And for at least 5 months after the last dose of experimental treatments.
14. All patients must understand and accept to comply with the conditions of the Lenalidomide pregnancy prevention plan (Appendix 18.4 of the protocol).

**FCBP: Female of Child Bearing Potential is any sexually mature female who:1) has achieved menarche at some point, 2) has not undergone a hysterectomy or bilateral oophorectomy or 3) has not been naturally postmenopausal (not having menstrual cycles due to cancer therapy does not rule out childbearing potential) for at least 24 consecutive months.*

### Exclusion criteria. Eligible patients

Any potential subject who meets any of the following criteria will be excluded from participating in the study.

1 Subject has a diagnosis of primary amyloidosis, monoclonal gammopathy of undetermined significance, or smoldering multiple myeloma. Monoclonal gammopathy of undetermined significance is defined by presence of serum M-protein <3 g/dL; absence of lytic bone lesions, anemia, hypercalcemia, and renal insufficiency related to the M-protein; and (if determined) proportion of plasma cells in the bone marrow of 10% or less (Kyle 2003). Smoldering multiple myeloma is defined as asymptomatic multiple myeloma with absence of related organ or tissue impairment end organ damage (Kyle 2003, Kyle 2007).

2 Subject has a diagnosis of Waldenström's disease, or other conditions in which IgM M-protein is present in the absence of a clonal plasma cell infiltration with lytic bone lesions.

3 Subject has prior or current systemic therapy or SCT for multiple myeloma, with the exception of an emergency use of a short course (equivalent of dexamethasone 40 mg/day for a maximum 4 days) of corticosteroids before treatment.

4 Subject has a history of malignancy (other than multiple myeloma) within 3 years before the date of randomization (exceptions are squamous and basal cell carcinomas of the skin and carcinoma in situ of the cervix, or malignancy that in the opinion of the investigator, with concurrence with the sponsor's medical monitor, is considered cured with minimal risk of recurrence within 3 years).

5 Subject has had radiation therapy within 7 days of randomization*

* unless done for antalgic reason or in case of functional risk for the patient.

6 Subject has had plasmapheresis within 7 days of randomization*

* *unless patient disease is still measurable (inclusion criteria n°6) after the plasmapheresis.*

7 Subject is exhibiting clinical signs of meningeal involvement of multiple myeloma.

8 Known to be seropositive for history of human immunodeficiency virus (HIV) or to have hepatitis A active infection.

9 Known to have hepatitis B active or uncontrolled infection (positive HBsAg and/or HBV DNA)

- Patient can be eligible if anti-HBc IgG positive (with or without positive anti-HBs) but HBsAg and HBV DNA are negative.

  If anti-HBV therapy in relation with prior infection was started before initiation of IMP, the anti-HBV therapy and monitoring should continue throughout the study treatment period.
- Patients with negative HBsAg and positive HBV DNA observed during screening period will be evaluated by a specialist for start of anti-viral treatment: study treatment could be proposed if HBV DNA becomes negative and all the other study criteria are still met.

10 Known to have hepatitis C active infection (positive HCV RNA and negative anti-HCV)

Patients with antiviral therapy for HCV started before initiation of IMP and positive HCV antibodies are eligible. The antiviral therapy for HCV should continue throughout the treatment period until seroconversion.

Patients with positive anti-HCV and undetectable HCV RNA without antiviral therapy for HCV are eligible.

11 Subject has any clinically significant medical or psychiatric condition or disease (e.g., uncontrolled diabetes, acute diffuse infiltrative pulmonary disease) in the investigator's opinion, would expose the patient to excessive risk or may interfere with compliance or interpretation of the study results.

12 Subject has active systemic infection and severe infections requiring treatment with a parenteral administration of antibiotics.

13 Subject has clinically significant cardiac disease, including:

- myocardial infarction within 6 months before randomization, or an unstable or uncontrolled disease/condition related to or affecting cardiac function (e.g., unstable angina, congestive heart failure, New York Heart Association Class III-IV)
- uncontrolled cardiac arrhythmia (National Cancer Institute Common Terminology Criteria for Adverse Events [NCI CTCAE] Version 5 Grade ≥2) or clinically significant ECG abnormalities or LVEF < 40 %

14. Subject has known allergies, hypersensitivity, or intolerance to steroids, mannitol, pregelatinized starch, sodium stearyl fumarate, histidine (as base and hydrochloride salt), arginine hydrochloride, poloxamer 188, sucrose or any of the other components of study intervention that are not amenable to premedication with steroids and H2 blockers or would prohibit further treatment with these agents, monoclonal antibodies or human proteins, or their excipients (refer to respective package inserts or Investigator's Brochure).
15. Known hypersensitivity, allergy to one of the study products (isatuximab, lenalidomide, bortezomib), dexamethasone or to one of the excipients.
16. Acute diffuse infiltrative pneumopathy, pericardial disease
17. Subject has plasma cell leukemia (according to World Health Organization [WHO] criterion: ≥20% of cells in the peripheral blood with an absolute plasma cell count of more than $2 \times 10^9$/L) or POEMS syndrome (polyneuropathy, organomegaly, endocrinopathy, monoclonal protein, and skin changes).
18. Subject is known or suspected of not being able to comply with the study protocol (e.g., because of alcoholism, drug dependency, or psychological disorder). Subject has any condition for which, in the opinion of the investigator, participation would not be in the best interest of the subject (e.g., compromise the well-being) or that could prevent, limit, or confound the protocol- specified assessments. Subject is taking any prohibited medications.
19. Subject has had major surgery within 2 weeks before randomization or has not fully recovered from surgery, Kyphoplasty or vertebroplasty is not considered major surgery.
20. Subject has received an investigational drug (including investigational vaccines) within 14 days or 5 half-lives of the investigational drug prior to initiation of study intervention, whichever is longer, or used an invasive investigational medical device within 4 weeks before randomization or is currently enrolled in an interventional investigational study.

    In case of very aggressive disease (i.e. circulating–plasma cell) delay could be shortened after agreement between sponsor and investigator, in absence of residual toxicities from previous therapy.
21. Refusal to consent or protected by legal regime (under judicial protection, guardianship, trusteeship).

22. Subject has contraindications to required prophylaxis for deep vein thrombosis and pulmonary embolism.
23. Incidence of gastrointestinal disease that may significantly alter the absorption of oral drugs.

NOTE: Investigators should ensure that all study enrolment criteria have been met at screening. If a subject's status changes (including laboratory results or receipt of additional medical records) after screening but before the first dose of study treatment is given such that he or she no longer meets all eligibility criteria, then the subject should be excluded from participation in the study.

Determination of the MRD marker at study entry is not a trial inclusion criterion

**Pre-injection Medications**

To decrease the risk and severity of infusion-related reactions (IR) to Isatuximab IV, which typically occur within 24 hours from the start of an infusion, and most commonly during the first infusion, all patients were to receive premedication administration in the following order, oral montelukast 10 mg orally 15 to 30 minutes (and never >60 minutes), dexamethasone 20 mg PO or IV (for patients who cannot tolerate dexamethasone during study treatment, methylprednisolone 100 mg IV or hydrocortisone hemisuccinate 50 or 100 mg IV can be administered as premedication only), acetaminophen 650 mg to 1000 mg PO (or equivalent), and then, diphenhydramine 25 mg to 50 mg IV or PO (or equivalent). Once the premedication regimen is completed, the isatuximab infusion must start immediately. Patients who do not experience an IR upon their first 4 administrations of isatuximab may have their need for subsequent premedication reconsidered.

However, both drugs cannot be used at the same time for premedication purposes.

Patients who do not experience an IAR upon their first 4 administrations of isatuximab may have their need for subsequent premedication reconsidered, at the Investigator's discretion.

The investigator should specify the number of consecutive infusions at which, if a patient does not experience an IR, the need for premedication can be considered no longer mandatory.

**Endpoints and Assessments**

The primary endpoint was Minimal Residual Disease (MRD) rate at 10-5 at 18 months.

The key secondary endpoints included in either arm, safety, various response assessments, various MRD assessments and survival endpoints to the treatments. Tumor response and disease progression were assessed in accordance with International Myeloma Working Group (IMWG) response criteria. Adverse events were monitored continuously from informed consent through 30 days past the last study treatment and graded according to National Cancer Institute Common Terminology Criteria for Adverse Events Version 5 (details in the Supplementary Appendix).

The endpoints were studied at in the specific population of patients with high risk multiple myeloma (HRMM).

**MRD assessment and method.**

The primary objective was to evaluate Minimal Residual Disease (MRD) rate at 10-5 in both arms at 18 months of randomization to Isa-VRd versus IsaRd in newly diagnosed NTE non-frail Multiple Myeloma patients.

MRD-negativity rate was defined as the overall proportion of patients who achieved MRD negativity (at or below a sensitivity threshold of $10^{-5}$) and in patients who achieved both MRD negativity (at or below a sensitivity threshold of $10^{-5}$) and a complete response or better at any time during the study after randomization, according to IMWG criteria[3].

MRD was performed on bone marrow aspiration/selected plasma cells in patients at least in ≥PR (≥50% reduction tumoral mass) for the primary endpoint timepoint, 18 months, and in patients ≥75% reduction tumoral mass for the other MRD timepoints (secondary endpoints). The analysis of MRD is done in ITT, the patients with primary refractory disease and patients with SD, along with patients failing MRD analysis, will be considered as patients with MRD positive at $10^{-5}$ at any time point.

The MRD test was centrally and primarily determined by next generation sequencing (NGS) with a $10^{-6}$ sensitivity (Pr Avet Loiseau / Pr Jill Corre, Toulouse Oncopole, France). In case of failure to perform MRD by NGS, MRD assessment was then performed centrally using multiparametric flow cytometry (MFC) with a $10^{-5}$ sensitivity (Pr Avet Loiseau / Pr Jill Corre, Toulouse Oncopole, France).

Two milliliters of fresh bone marrow were sent overnight to Oncopole Toulouse, France. Upon receipt, bone marrow cells were counted, followed by a standard red blood cell lysis. Two to three million of cells were kept for MFC analysis, the remaining cells were used for NGS analysis.

Next Generation Sequencing was performed using the Clonoseq 2.0™ kit (under an agreement with Adaptive Biotechnologies, Seattle, USA). Limit of Detection (LOD) and MRD status were determined using Adaptive's validated algorithms for the clonoSEQ V2.0 assay.

Multiparametric flow cytometry has been performed according to the Euroflow recommendations. Briefly, the bone marrow (BM) samples were processed after red blood cell lysis (VersaLyse, Beckman Coulter, Brea, CA) and phosphate-buffered saline washing. Surface antigens and cytoplasmic immunoglobulin light chain staining was then performed after IntraStain (Dako, Glostrup, Denmark) cell permeabilization.

For identification of BM Plasma cells (PCs) and discrimination between phenotypically aberrant and normal PCs, two-tubes/eight-colors of antibodies panels were used: tube 1: CD138-BV421,CD27-BV510, CD38-FITC, CD56-PE, CD45-PerCPCy5.5, CD19-PECy7, CD117-APC, CD81-APCH7 and; tube 2: CD138-BV421, CD27-BV510, CD38-FITC,CD56-PE, CD45-PerCPCy5.5, CD19-PECy7, cyKAPPA-APC, cyLAMBDAAPCH7*)*[4]Data were acquired on a FACSCanto II flow cytometer equipped with FACSDiva version 6.2 data acquisition and analysis software (BD Biosciences). The analysis of all plasma cell subsets was performed as reported[5,6]. To detect MRD at a sensitivity of at

least $10^{-5}$, the minimum events of total nucleated cells acquired and required was $2 \times 10^{6}$ (or more) with a minimum neoplastic population size of 20 events.

At 25/03/2024, 223 patients have done the MRD assessment test at 18 months. The following table summarizes the repartition between the MRD tests performed using NGS assessment, and when failing the MRD tests performed using MFC assessment.

| **n (%)** | **All** | **IsaRd** | **Isa-VRd** |
|---|---|---|---|
| **NGS** | 221 (99) | 109 (98) | 112 (100) |
| **MFC** | 2 (1) | 2 (2) | 0 |

NGS. Next Generation Sequencing; MFC. Multiparametric flow cytometry. N. number.

**The secondary objectives.**

Secondary objectives included to determine safety in either arm, studying clinical and laboratory parameters, adverse events, and vital signs according to CTCAE 5.0.

The secondary objectives also included to determine the best response to the treatment and at 6, 12, 18 and yearly in either arm according to the International Myeloma Working Group (IMWG)[3] overall rates of overall response, very good partial response or better, and complete response or better were defined as the percentage of patients in the intention-to-treat population who achieved partial response or better status, very good partial response or better status, and complete response status, respectively, at any time during the study per International Myeloma Working Group criteria. In addition, the specific response must have been achieved prior to the start of subsequent therapies. It was also planned to determine time to first response, time to VGPR, and time to best response: defined as the time from randomization to the date of first documentation of PR, VGPR, first documentation of CR. Response duration to both regimens for responders, defined as the time from first response (≥PR) to the date of first documentation of progression. The rate of primary refractory patients, defined as the proportion of patients with SD or DP as assessed by the by Investigator.

The secondary objectives also included various MRD assessments, including the MRD negative rate at 12 months, and yearly, at the $10^{-5}$ and at the $10^{-6}$ thresholds, post randomization (and prior to disease progression, receipt of subsequent therapy, or both), defined as the proportion of patients with MRD in bone marrow aspirate ($< 10^{-5}$), according to IMWG criteria for patient who reached at least ≥75% reduction tumoral mass (or for patient who has reached at least SD and with MRD negative at the previous MRD assessment). In ITT, it is considered that primary refractory patients, patients with SD and minor response will be considered as patients with MRD positive at $10^{-5}$.

The sustained MRD rate at $10^{-5}$ (similar time points 12 and 24 months), defined as the proportion of patients with sustained MRD in bone marrow aspirate ($< 10^{-5}$) between 2 evaluations was determined in the protocol. We studied the rate of loss of MRD at $10^{-5}$ at each time point defined as the proportion

of patients with MRD negative at $10^{-5}$ who lose the MRD negative status at the next evaluation. We also studied the time to reach MRD negative rate at $10^{-5}$ and loss of MRD negative rate. at $10^{-5}$, defined as the time from randomization to the date of the first MRD negative rate at $10^{-5}$ and the time from randomization or from the date of MRD negative at $10^{-5}$ to the date of MRD positive at $10^{-5}$, respectively. Finally, we planned to study the duration of MRD negativity, defined as the time from the date of first documentation of minimal residual disease negativity to the date of first documentation of confirmed disease progression, death due to disease progression, or loss of MRD negativity (at $10^{-4}$ or higher), whichever occurred first, for patients who achieved MRD negativity in the study. Patients without disease progression or loss of MRD-negative status were censored at the last disease evaluation prior to subsequent therapy or the date of last MRD negativity, whichever was later. Sustained MRD negativity lasting ≥12 months was defined as two consecutive MRD-negative results ($10^{-5}$) at least 12 months apart, without any MRD-positive results in between.

The following survival endpoints were studied as secondary endpoints in either arm, Overall Survival (OS), Progression free survival (PFS), Time to Progression (TTP), Time to Next Therapy (TTNT) and Event Free survival (EFS). Progression-free survival was defined as the time from the date of randomization to the date of first disease progression according to the International Myeloma Working Group response criteria or death due to any cause, whichever occurred earlier. Overall survival was measured from the date of randomization to the date of death due to any cause. Patients who were lost to follow-up were censored at the time that they were lost to follow-up. Patients who died after consent withdrawal were considered as having an overall survival event. Patients who were still alive at the clinical cutoff date for the analysis were censored at the last known date that they were alive; this date was determined by the maximum collection/assessment date from among selected data domains within the clinical database. The time to progression was defined as the time from randomization to the date of first documentation of DP (as determined by the investigator using IMWG criteria). The time to next treatment was defined as time from discontinuation from treatment to the date of next myeloma therapy in patients that had progression and are alive. Study of TTNT will be done from study entry to next therapy. The event free survival was defined as permanent discontinuation of study treatment as a whole, death or progression whichever occurs first. Study of EFS will be done from study entry to event, whichever occurs first.

There has been a series of exploratory objectives collected within the study frame and listed as follow, to determine molecular signature of “excellent responders” and “extremely poor responders” in either arm, to study sFLC escape in either arm, to evaluate the respective incidence rate of sflc normalized ratio versus MRD rate, to evaluate the incidence rate of patients CR and MRD negative but remaining PET CT positive, and its prognostic impact, to compare MRD blood versus marrow, and to study the impact of ISS (B2-microglobulin, albumin) and R ISS (B2-microglobulin, albumin, LDH, chromosomal abnormalities).

**Cytogenetic risk assessment.**

Cytogenetic risk was assessed according to Perrot et al. for study analysis[2].

Randomization was stratified by age (< 75 and ≥ 75) and cytogenetic risk at baseline. For this purpose and to limit bias in recruitment, cytogenetic risk assessment was performed by FISH (Modified Perrot score) using probes for chromosomes 17, 14, 4, and 1.

All patients had also cytogenetic risk assessed by NGS according to Perrot et al. used for study analysis. Cytogenetic risk was assessed according to Perrot et al. for study analysis. Bone marrow samples were obtained at diagnosis and shipped overnight to a central laboratory. Upon receipt, plasma cells (PCs) were isolated using CD138+ MAC-Sorting (Miltenyi Biotec, Paris, France). Post-sorting purity was checked by cytologic analysis of a spin from positive fraction, and only samples with ≥ 70% PCs after sorting were kept for the analysis. The mean purity was 94%. PCs were analyzed by NGS using NextSeq 500 (Illumina). For each positive del(17p) by NGS, an additional FISH analysis was performed to assess the percentage of positive plasma cells. NGS sequencing was performed using a panel of specific probes targeting regions of interest, as previously described[7].

Adapted Perrot et al. score2.

| Abnormality | Coefficient |
|---|---|
| Trisomy 5 | -0,3 |
| Trisomy 21 | 0,3 |
| T(4 ;14) | 0,4 |
| Gain 1q | 0,5 |
| Del(1p32) | 0,8 |
| Del17p | 1,2 |
| **LP score** = 0.4 x t(4;14) + 1.2 x del17p – 0.3 x tri5 + 0.3 x tri21 + 0.5 x gain1q + 0.8 x del1p32 | |

| **Modified LP score** = 0.4 x t(4;14) + 1.2 x del17p + 0.5 x gain1q + 0.8 x del1p32 | |
|---|---|
| **LP score / Modified PL score** | **Risk** |
| **≤ 0** (OS 5y>75%) | Low |
| **0 < LP ≤ 1**(50%<OS 5y<75%) | Medium |
| **LP > 1**(OS 5y<50%) | High |

## Cytogenetic risk determined by Fish

| | IsaRd (N=135) | Isa-VRd (N=135) |
|---|---|---|
| **LP score** | | |
| **n** | 128 | 130 |
| **LP score** | 0 [0;0.5] (0;1.7) | 0 [0;0.5] (0;1.7) |
| **Cytogenetic profile (strata)** | | |
| **Per-** | 124 (92%) | 119 (88%) |
| **Per+** | 11 (8%) | 16 (12%) |
| **t(4;14)** | | |
| **No** | 120 (92%) | 120 (92%) |
| **Yes** | 10 (8%) | 10 (8%) |
| **del(17p)** | | |
| **No** | 125 (96%) | 122 (94%) |
| **Yes** | 5 (4%) | 8 (6%) |
| **del(17p) (%)** | 83 [72;90] (68;93) | 78.5 [72.8;81.8] (63;92) |
| **del(1p32)** | | |
| **No** | 119 (92%) | 117 (90%) |
| **Yes** | 10 (8%) | 13 (10%) |
| **1q gain** | | |
| **No** | 91 (71%) | 82 (63%) |
| **Yes** | 38 (29%) | 48 (37%) |

## Cytogenetic risk determined by NGS

| | IsaRd (N=135) | Isa-VRd (N=135) |
|---|---|---|
| **LP score** | | |
| **n** | 126 | 129 |
| **LP score** | 0 [0;0.5] (-0.3;1.7) | 0 [0;0.5] (-0.3;1.4) |
| **Cytogenetic profile (strata)** | | |
| **Favourable risk (LP ≤ 0)** | 75 (60%) | 68 (53%) |
| **Intermediate risk (LP in ]0 ; 1])** | 41 (33%) | 48 (37%) |
| **High risk (LP > 1)** | 10 (8%) | 13 (10%) |
| **t(4;14)** | | |
| **No** | 117 (92%) | 119 (92%) |

| | | |
|---|---|---|
| **Yes** | 10 (8%) | 10 (8%) |
| **del(17p)** | | |
| **No** | 122 (96%) | 121 (94%) |
| **Yes** | 5 (4%) | 8 (6%) |
| **del(17p) (20% cutoff)** | 8 (6%) | 11 (8%) |
| **del(17p) (50% cutoff)** | 6 (5%) | 8 (65) |
| **del(1p32)** | | |
| **No** | 117 (92%) | 115 (89%) |
| **Yes** | 10 (8%) | 14 (11%) |
| **1q gain** | | |
| **No** | 88 (70%) | 84 (65%) |
| **Yes** | 38 (30%) | 45 (35%) |
| **1q gain** | 38 (30%) | 45 (35%) |
| **1q amplification** | 7 (6%) | 12 (9%) |
| **t(14;16)** | | |
| **No** | 125 (98%) | 125 (97%) |
| **Yes** | 2 (2%) | 4 (3%) |
| **Trisomy 5** | | |
| **No** | 65 (51%) | 62 (48%) |
| **Yes** | 62 (49%) | 67 (52%) |
| **Trisomy 21** | | |
| **No** | 98 (77%) | 91 (71%) |
| **Yes** | 29 (23%) | 38 (29%) |

**Statistical Analysis**

Assuming that 15% of patients would be MRD negative at 18 months in the IsaRd arm (based on approximated initial results from the MAIA trial), inclusion of 242 patients would give an 80% power to detect an improvement from 15% to 30% in the Isa-VRd arm at a 2-sided α of 0.05. To account for potential dropouts, 270 patients were planned to be enrolled. The primary analysis was performed in the intent-to-treat population, which included all randomized patients. The safety population included all patients who had received at least one dose of the assigned treatment.

The primary endpoint was compared between treatment groups and treatment effect was assessed by odds ratio and 95% confidence interval using a mixed logistic regression with treatment as the explanatory variable and adjusting for randomization stratification factors, cytogenetic risk (high risk

vs. intermediate or low risk) stage (>75 yo. Vs. <= 75 yo.) as fixed effect and type of center as random effect. All other binary secondary endpoints were analyzed similarly to the primary endpoint.

For time-to-event endpoints, distribution was estimated and plotted using Kaplan-Meier method (Progression-Free Survival, Overall Survival), or Gray cumulative incidence method in case of competition (time to first response, time to best response). Endpoints were compared between arm, and treatment effect was assessed by Hazard Ratio (or cause-specific Hazard Ratio in case of competing risks) and 95% confidence interval using a Cox proportional Hazard model with treatment as explanatory variable and adjusting for randomization stratification factors. When data were not mature enough, no test was performed and no hazard ratio was estimated.

Homogeneity of treatment effect on the primary endpoint was checked by plotting effect in predefined subgroups using a Forest plot and testing for significance of an interaction term included in a logistic regression model.

No interim analysis was planned and all endpoints were tested at a two-sided alpha level of 5% without correction for multiplicity.

**Data and Safety Monitoring Board (DSMB).**

The Data and Safety Monitoring Board independent consultative committee was responsible for reviewing the data and safety of the study. The DSMB consisted of 2 medical doctors, one expert in hematology but not Myeloma and one expert in Myeloma not involved in the study protocol and one methodologist not involved with study design and statistical calculations. We want to thank the members of the independent data and safety monitoring committee, Pr Jean-Paul Fermand, chair, Pr Nathalie Meuleman (Belgium), Pr Stephanie Ragot (methodologist). The DSMB met every 6 months for safety analysis (AE grade ≥ 3 and SAE), and treatment discontinuation reasons.

**Supplemental appendix Figure 1. Trial Design.**

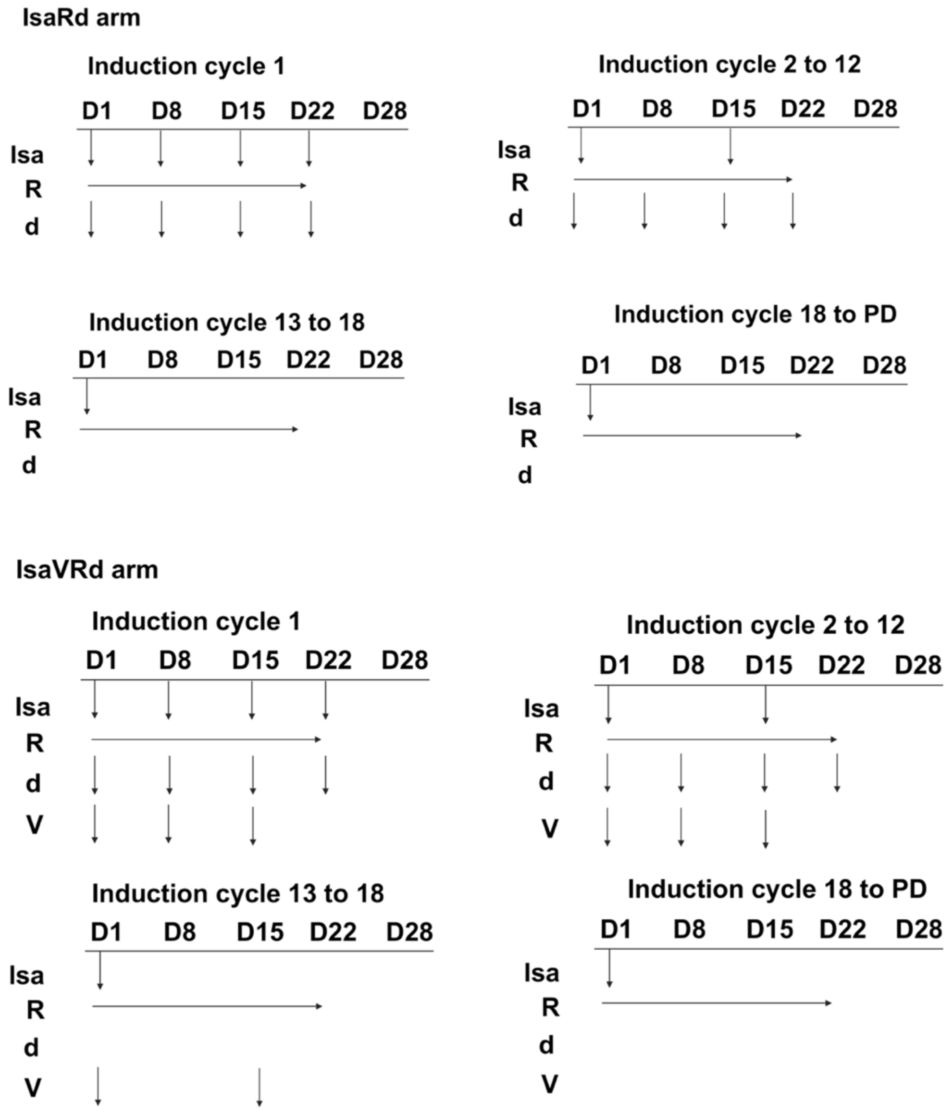


IsaRd denotes isatuximab/lenalidomide/dexamethasone.

Isa-VRd denotes isatuximab plus bortezomib/lenalidomide/dexamethasone.

R, lenalidomide. Isa, isatuximab. D, dexamethasone. V, bortezomib. PD, progressive disease. D, day.

**References**


1. Rajkumar SV, Dimopoulos MA, Palumbo A, et al. International Myeloma Working Group updated criteria for the diagnosis of multiple myeloma. Lancet Oncol. 2014
2. Perrot A, Lauwers-Cances V, Tournay E, et al. Development and validation of a cytogenetic prognostic index predicting survival in multiple myeloma. J Clin Oncol 2019
3. Kumar S, Paiva B, Anderson KC, et al. International Myeloma Working Group consensus criteria for response and minimal residual disease assessment in multiple myeloma. Lancet Oncol. 2016
4. Arana P, Paiva B, Cedena MT, et al. Prognostic value of antigen expression in multiple myeloma: A PETHEMA/GEM study on 1265 patients enrolled in four consecutive clinical trials. Leukemia 2018
5. Robillard N, Béné MC, Moreau P, Wuillème S. A single-tube multiparameter seven-colour flow cytometry strategy for the detection of malignant plasma cells in multiple myeloma. Blood Cancer J. 2013
6. Wuillème S, Lok A, Robillard N, et al. Assessment of tumoral plasma cells in apheresis products for autologous stem cell transplantation in multiple myeloma. Bone Marrow Transplant. 2016
7. Bolli N, Biancon G, Moarii M, et al. Analysis of the genomic landscape of multiple myeloma highlights novel prognostic markers and disease subgroups. Leukemia 2018
