# Supplementary material for: Isatuximab, lenalidomide, dexamethasone and bortezomib in transplant-ineligible multiple myeloma: the randomized phase 3 BENEFIT trial
Source: Nat Med. 2024 Jun 3;30(8):2235–41. doi: 10.1038/s41591-024-03050-2 (PMC11333283; doi:10.1038/s41591-024-03050-2)
Supplement: Supplementary file 2 — Reporting Summary [file 41591_2024_3050_MOESM2_ESM.pdf]

nature portfolio

Corresponding author(s): Xavier Leleu

Last updated by author(s): 19 April, 2024

Reporting Summary

Nature Portfolio wishes to improve the reproducibility of the work that we publish. This form provides structure for consistency and transparency in reporting. For further information on Nature Portfolio policies, see our Editorial Policies and the Editorial Policy Checklist.

Statistics

For all statistical analyses, confirm that the following items are present in the figure legend, table legend, main text, or Methods section.

n/a	Confirmed	
☐	☒	The exact sample size (n) for each experimental group/condition, given as a discrete number and unit of measurement
☐	☒	A statement on whether measurements were taken from distinct samples or whether the same sample was measured repeatedly
☐	☒	The statistical test(s) used AND whether they are one- or two-sided<br>*Only common tests should be described solely by name; describe more complex techniques in the Methods section.*
☐	☒	A description of all covariates tested
☐	☒	A description of any assumptions or corrections, such as tests of normality and adjustment for multiple comparisons
☐	☒	A full description of the statistical parameters including central tendency (e.g. means) or other basic estimates (e.g. regression coefficient) AND variation (e.g. standard deviation) or associated estimates of uncertainty (e.g. confidence intervals)
☐	☒	For null hypothesis testing, the test statistic (e.g. F, t, r) with confidence intervals, effect sizes, degrees of freedom and P value noted<br>*Give P values as exact values whenever suitable.*
☒	☐	For Bayesian analysis, information on the choice of priors and Markov chain Monte Carlo settings
☒	☐	For hierarchical and complex designs, identification of the appropriate level for tests and full reporting of outcomes
☒	☐	Estimates of effect sizes (e.g. Cohen's d, Pearson's r), indicating how they were calculated

Our web collection on statistics for biologists contains articles on many of the points above.

Software and code

Policy information about availability of computer code

Data collection	V8.2.30 Ennov
Data analysis	All statistical analyses were performed using R software version 4.2.1

For manuscripts utilizing custom algorithms or software that are central to the research but not yet described in published literature, software must be made available to editors and reviewers. We strongly encourage code deposition in a community repository (e.g. GitHub). See the Nature Portfolio guidelines for submitting code & software for further information.

Data

Policy information about availability of data

All manuscripts must include a data availability statement. This statement should provide the following information, where applicable:

- Accession codes, unique identifiers, or web links for publicly available datasets
- A description of any restrictions on data availability
- For clinical datasets or third party data, please ensure that the statement adheres to our policy

Data supporting this article are part of an ongoing clinical trial and are not publicly available. Data will be considered for sharing once the product and indication has been approved by major health authorities (for example, the US Food & Drug Administration, the European Medicines Agency), with restriction due to data privacy regulations, and the informed consent.
Requests for de-identified patient data by researchers with proposed use of the data can be made to the corresponding author with specific data needs, analysis

plans and dissemination plans. Those requests will be reviewed by a study steering committee and the study sponsor for release upon publication. Response will typically be given in 3 months.
The trial protocol and statistical analysis plan can be found in the Supplementary Information.

Human research participants

Policy information about studies involving human research participants and Sex and Gender in Research.

Reporting on sex and gender	Sex was collected and reported in the trial
Population characteristics	Eligible patients had NDMM TI, aged of 65 to 79 years, summarized in the Supplementary Appendix and Table 1.
Recruitment	This open-label, multicenter parallel arms phase 3 trial randomly assigned patients between 07, September 2021 and 02, September 2022, recruited across 60 centers in France. Eligible patients had NDMM TI, aged of 65 to 79 years (Supplementary Appendix).Patients were randomly (1:1 ratio) assigned to Isa-VRd or IsaRd until progression, one cycle being 28 days long. Randomization was stratified by age (< 75 and ≥ 75), cytogenetic risk at baseline assessed by fluorescence in situ hybridization (Supplementary Appendix) and type of centre (based on volume and teaching status). There has been no selection of patients.
Ethics oversight	An independent French ethics committee approved the study protocol, alongside ANSM. The informations on the ethic committee and the communications with the sponsor are available upon request. All patients provided informed consent.

Note that full information on the approval of the study protocol must also be provided in the manuscript.

Field-specific reporting

Please select the one below that is the best fit for your research. If you are not sure, read the appropriate sections before making your selection.

☒ Life sciences ☐ Behavioural & social sciences ☐ Ecological, evolutionary & environmental sciences

For a reference copy of the document with all sections, see nature.com/documents/nr-reporting-summary-flat.pdf

Life sciences study design

All studies must disclose on these points even when the disclosure is negative.

Sample size	The sample size is summarized in the chapter Statistical Analysis of the manuscript, it is also summarized in the Supplemntal Appendix. Statistical Analysis. Assuming that 15% of patients would be MRD negative at 18 months in the IsaRd arm (based on approximated initial results from MAIA), inclusion of 242 patients would give an 80% power to detect an improvement from 15% to 30% in the Isa-VRd arm at a 2-sided α of 0.05. To account for potential dropouts, 270 patients were planned to be enrolled.
Data exclusions	No data exclusions was performed
Replication	NA (clinical trial)
Randomization	Patients were randomly (1:1 ratio) assigned to each treatement arm. Randomization was stratified by age (< 75 and ≥ 75), cytogenetic risk at baseline assessed by fluorescence in situ hybridization (Supplementary Appendix) and type of centre (based on volume and teaching status).
Blinding	This was an open-label trial. Primary outcome (MRD by NGS at month 18) was centrally assessed by a team blinded to the treatment arm which minimize the risk of measurement bias. For this reason, blinding, which would have required the use of a subcutaneous placebo of bortezomib in the control arm for 18 months was considered an unnecessary burden for both the patients in control arm and the trial organization.

Reporting for specific materials, systems and methods

We require information from authors about some types of materials, experimental systems and methods used in many studies. Here, indicate whether each material, system or method listed is relevant to your study. If you are not sure if a list item applies to your research, read the appropriate section before selecting a response.

Materials & experimental systems

n/a	Involved in the study
☒	☐ Antibodies
☒	☐ Eukaryotic cell lines
☒	☐ Palaeontology and archaeology
☒	☐ Animals and other organisms
☐	☒ Clinical data
☒	☐ Dual use research of concern

Methods

n/a	Involved in the study
☒	☐ ChIP-seq
☒	☐ Flow cytometry
☒	☐ MRI-based neuroimaging

Clinical data

Policy information about clinical studies
All manuscripts should comply with the ICMJE guidelines for publication of clinical research and a completed CONSORT checklist must be included with all submissions.

Clinical trial registration

NCT04751877

Study protocol

The full trial protocol can be accessed upon request along with all amendments made to the protocol, and the first and last versions of the protocol.

Data collection

The patients were recruited between 07, September 2021 and 02, September 2022, recruited across 60 centers in France. The IFM and CHU Poitiers in collaboration with the investigators, designed the trial and compiled and maintained the data collected by the investigators throughout the study until the clinical cutoff date (25 March, 2024). The full list of participating centers, investigators and staffs is available in the supplementary appendix. The eCRF version is the V8.2.30 Ennov CRF.

Outcomes

The primary endpoint was minimal residual disease (MRD) rate at or below a sensitivity threshold of 10–5 at 18 months from randomization.
MRD was performed on bone marrow aspiration in patients who achieved at least a partial response (≥PR) for the primary endpoint timepoint of 18 months. The patients with primary refractory disease, stable disease and minor response, along with patients failing or not tested for MRD analysis, have been considered as patients with positive MRD at 10-5. The MRD test was centrally and primarily determined by next generation sequencing (NGS) with a 10-6 sensitivity (Professor Avet Loiseau / Professor Corre, Toulouse Oncopole, France). In the case of failure to perform MRD by NGS, MRD assessment was then performed centrally using multiparametric flow cytometry (MFC) with a 10-5 sensitivity (Dr Vergez, Toulouse Oncopole, France) (see Supplementary Appendix).
Key secondary endpoints included were response assessments, MRD at different time points and different thresholds, Progression Free Survival, Overall Survival. and Safety
Response was defined according to International Myeloma Working Group criteria
Progression-free survival was defined as the time from the date of randomization to the earlier date between date of first disease progression according to the International Myeloma Working Group response criteria or death due to any cause
Overall survival was measured from the date of randomization to the date of death due to any cause
For safety, all advrese events (AE) are recorded in standard medical terminology and graded according to the National Cancer Institute Common Terminology Criteria for Adverse Events (NCI-CTCAE), Version 4.03. For AE reporting, the verbatim term used in the CRF by investigators to identify adverse events is coded using the latest version of Medical Dictionary for Regulatory Activities (MedDRA) coding dictionary. Treatment-emergent adverse events (TEAEs) are defined as any AE that occurs after start of the first study treatment through 30 days after the last study treatment; or the day prior to start of subsequent anti-myeloma therapy, whichever is earlier; or any AE that is considered drug-related (very likely, probably, or possibly related) regardless of the start date of the event; or any AE that is present at baseline but worsens in toxicity grade or is subsequently considered drug-related by the investigator.
All endpoints are defined in the SAP

nature portfolio | reporting summary
